# Quercetin mitigates iron-induced cell death in chicken granulosa cell

**DOI:** 10.1186/s40104-024-01118-0

**Published:** 2024-12-08

**Authors:** Shuo Wei, Felix Kwame Amevor, Xiaxia Du, Linxiang Li, Zhixin Yi, Gang Shu, Yan Wang, Xiaoling Zhao

**Affiliations:** 1 State Key Laboratory of Swine and Poultry Breeding Industry, College of Animal Science and Technology, Sichuan Agricultural University, Chengdu, Sichuan P. R. China; 2 Farm Animal Genetic Resources Exploration and Innovation Key Laboratory of Sichuan Province, College of Animal Science and Technology, Sichuan Agricultural University, Chengdu, Sichuan 611130 China; 3 Key Laboratory of Livestock and Poultry Multi-omics, Ministry of Agriculture and Rural Affairs, Sichuan Agricultural University, Chengdu, Sichuan P. R. China; 4 Bazhong Academy of Agriculture and Forestry Sciences, Bazhong, P. R. China; 5 Department of Basic Veterinary Medicine, Sichuan Agricultural University, Chengdu, Sichuan P. R. China

**Keywords:** Apoptosis, Cellular inflammation, Ferroptosis, Granulosa cell, Quercetin

## Abstract

**Background:**

Granulosa cell (GC) apoptosis, ferroptosis, and other programmed cell death processes are markers of follicular aging. Quercetin has been shown to reduce ferroptosis, however, its effects on ferroptosis in poultry remains unexplored. Our preliminary study identified ferroptosis in aging ovaries. Therefore, in the present study, 540-day-old Mountain Plum-blossom chickens were fed with quercetin supplementation at varying doses (0.2, 0.4, and 0.6 g/kg), and examined its molecular effects on GC ferroptosis using an in vitro Erastin-induced model.

**Results:**

The results showed that quercetin supplementation significantly increased egg production, which confirmed its potential to alleviate ferroptosis in chicken ovarian tissue. The in vitro experiment revealed that quercetin and Fer-1 (positive control) mitigated Erastin-induced ferroptosis in GCs. Further, transcriptome analysis revealed that quercetin modulated key genes such as acyl-CoA synthetase long-chain family member 4 (*ACSL4*), solute carrier family 7 member 11 (*SLC7A11*), and transferrin receptor (*TFRC*), involved in ferroptosis regulation. The results further showed that quercetin also reduced Erastin-induced apoptosis and inflammation by modulating the expression of genes and proteins related to apoptosis and inflammatory factors (NF-κB, TNF-α, IL-6, and IL-10).

**Conclusion:**

Taken together, the results showed that quercetin improves egg production performance in chickens and mitigates ovarian ferroptosis in aging hens, and inhibits Erastin-induced ferroptosis, inflammation, and apoptosis in GCs. These findings revealed the protective role of quercetin in poultry ovarian tissue and its cellular mechanisms against detrimental factors in poultry production.

**Graphical Abstract:**

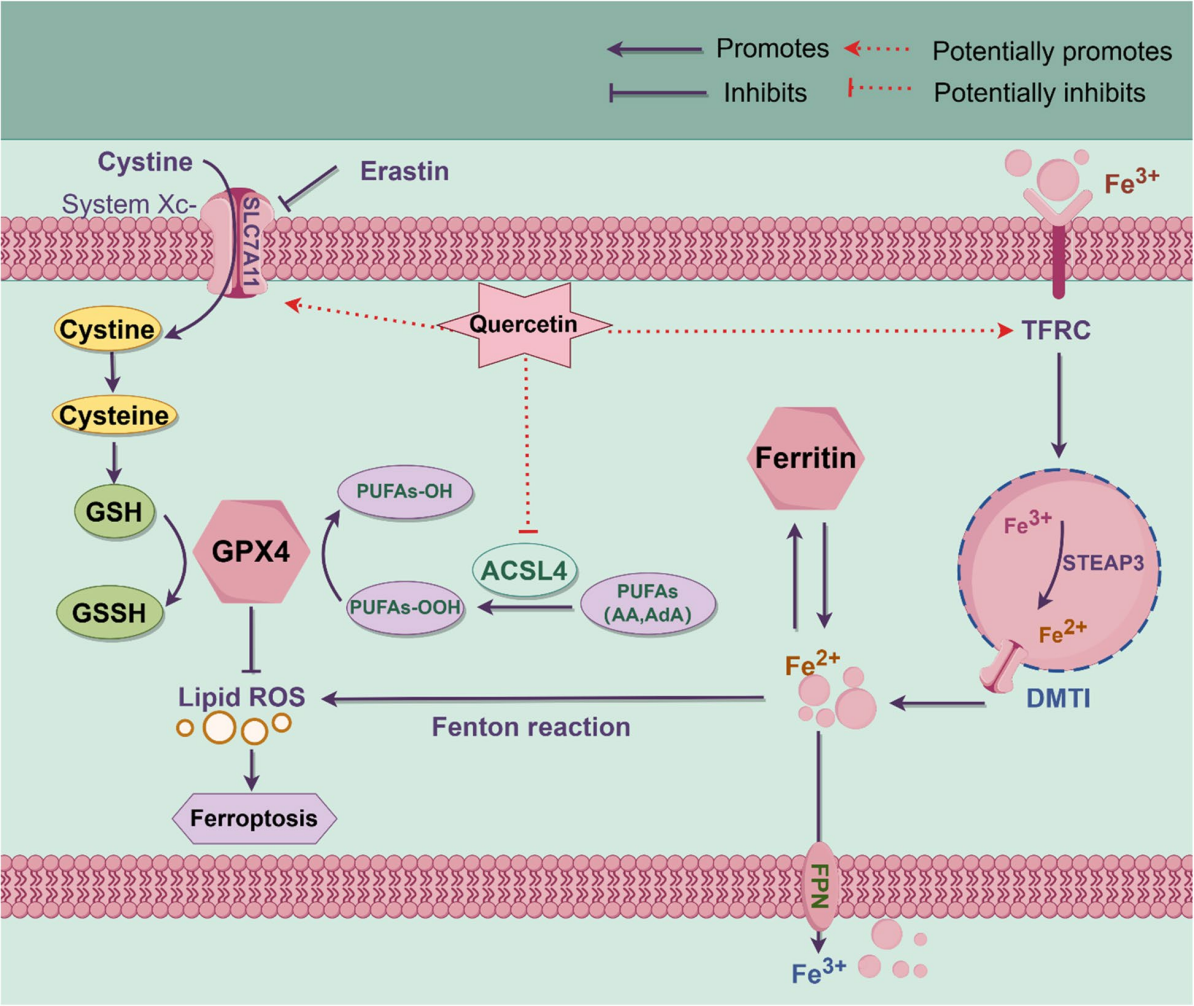

**Supplementary Information:**

The online version contains supplementary material available at 10.1186/s40104-024-01118-0.

## Introduction

It is widely recognized that egg production and quality in laying hens significantly decline during the late laying period [1]. Aging follicles in chickens can lead to delayed difficult ovulation, as well as follicular atresia. Studies have demonstrated that granulosa cell apoptosis is the trademark for the occurrence of follicle atresia [2]. However, apoptosis is not the sole factor contributing to this condition [3, 4]. Studies have showed that ferroptosis occurs in atretic follicles of pigs and geese [5, 6]. The concept of ferroptosis was first introduced by Professor Brent Stockwell from Columbia University, unveiling a cell death process that is neither apoptotic nor necrotic. This process is highly dependent on iron ions [7]. Subsequently, several studies have researched into the mechanism regulating ferroptosis, especially its potential role in neurodegenerative diseases, cancer treatment, cardiovascular diseases, and various inflammatory responses [8–10]. The main biomarkers of ferroptosis include a significant increase in the mass of lipid peroxides catalyzed by iron ions and the loss of function of the antioxidant defence system, such as glutathione and its related enzyme glutathione peroxidase 4 (GPX4) [11]. Erastin is a selective inducer of ferroptosis that inhibits the glutamate-cystine exchange transporter (System Xc-) on the cell membrane, leading to lipid peroxidation and ferroptosis. In contrast, ferrostatin-1 is a specific ferroptosis inhibitor that neutralizes intracellular lipid peroxides, thereby preventing ferroptosis.

Aging in both animals and humans leads to a significantly decline in ovarian and liver function, accompanied by hormonal and endocrine changes, reduced antioxidant capacity, and a decrease in follicle numbers. Previously our research group discovered a significant accumulation of lipid droplets in the atretic follicles of aging chickens, along with concentrated mitochondrial membrane density, reduced or absent mitochondrial cristae, and ruptured outer mitochondrial membranes, showing typical ferroptotic morphological characteristics compared to non-atretic follicular granulosa cells (GCs) as seen in Fig. S1. At the same time, we found that the iron content in the ovaries of chickens with atretic follicles was significantly higher than that in the non-atretic group as shown in Fig. S2. Therefore, we speculate that ferroptosis occurs in the atretic ovaries of nest chickens, which may be related to ovarian aging and atresia.

Quercetin is a yellow crystalline substance, a bioflavonoid with anti-inflammatory, antioxidant, anticancer, and antiviral properties. It is mainly found in a wide variety of vegetables and fruits in the form of glycosides. Quercetin has an excellent pharmacological benefit and is it natural and non-toxic, hence, it is widely used as a food additive in the field of food science, medicine, and animal husbandry [12–14]. Quercetin is considered a potent antioxidant because it has the ability to scavenge free radicals and bind metal ions [15]. Abnormal metabolism of iron ions in cells produce free radicals through Fenton reaction, thereby activates cellular oxidative stress which is the main factor for ferroptosis in cells. Studies indicated that quercetin forms stable chelates with metal ions, such as iron [16] and copper [17], to regulate their metabolism. Further, quercetin has been reported to alleviate ferroptosis in rat and human cancer cell lines [18], however, no study have been conducted in poultry.

In poultry production, quercetin has been shown to improve egg quality, enhance chicken growth performance, and boost immunity, making it a safe and multifunctional feed additive for use in poultry production [19]. At present, no studies have been reported on the effects of quercetin on ferroptosis in chickens. Most studies on quercetin remained at the individual level, however, its molecular mechanism of action at the cellular level has not yet been investigated. Therefore, exploring the protective effects of quercetin on GCs from ferroptosis at the cellular level provides theoretical basis for further investigation into follicular atresia in poultry. The addition of quercetin to inhibit ferroptosis, thereby protecting GCs and improving follicular atresia in laying hens during the later stages of egg production, holds significant scientific and practical potential.

## Materials and methods

### Animals and sample collection

A total of 240 (540 days old) Mountain Plum-blossom chickens in their late laying period obtained from the Mountain Plum-blossom chickens original breeding Unit (Tongjiang County, Bazhong City, China) were used in this study. The 240 Mountain Plum-blossom chickens with uniform body weight and similar egg production rate were randomly selected and allotted into four experimental groups, with four replicates per group and 15 chickens per replicate. Based on the amount of quercetin (99% purity, purchased from Shaanxi Huike Botanical Development Co., Ltd., Xi’an, China) added to the basal diet, the groups were divided into the control (0 g/kg), 0.2 g/kg, 0.4 g/kg, and 0.6 g/kg experimental groups. The control group was fed a basal diet, with each chicken receiving 100 g of feed daily, under 16 h of light, with drinking water provided ad libitum. The chicken pen was cleaned regularly, disinfected, and provided with natural ventilation. The composition and nutritional level of the basal diet are shown in the Table S1. After feeding for a period of 8 weeks, 6 chickens were randomly selected from each group. Then the selected chickens were euthanized by exsanguination via the oral cavity, and their ovaries were promptly and carefully excised. Then ovarian tissue samples were rinsed with phosphate-buffered saline (PBS), quickly frozen in liquid nitrogen, and finally stored at −80 °C for further analysis.

### Determination of biochemical indices and iron ion testing

Glutathione (GSH, Elabscience, Wuhan, China), total antioxidant capacity (T-AOC, Elabscience, Wuhan, China), superoxide dismutase (SOD, Elabscience, Wuhan, China), lipid peroxidation (LPO, Elabscience, Wuhan, China), reactive oxygen species (ROS, Mlbio, Shanghai, China) and the iron levels in the ovarian tissues of the late-stage laying hens were determined using the biochemical kits and an iron assay kit (Elabscience, Wuhan, China), following the instructions provided by the manufacturers.

### Cell culture and drug treatment

All the preovulatory follicles were picked from the ovaries and placed in a sterile Hank’s balanced salt solution (HBSS). The granular layer of the follicles was separated [20]. Furthermore, the granular layer was digested with β-II collagenase (BIO-FROXX, Einhausen, Germany), filtered with 70 µmol/L cell sieves, and then resus pended in Dulbecco’s Modified Eagle Medium/Nutrient Mixture F-12 (DMEM/F12) + 10% fetal bovine serum (Gibco, Grand Island, NY, USA). Thereafter, the GCs were cultured in an incubator at 37 °C, 5% CO_2_, and 95% air saturated humidity for cell attachment. Then the medium was changed to remove non-adherent cells after 3 h. Further, all the cells were cultured in the cell culture incubator at 37 °C, 5% CO_2_, and 95% air saturated humidity, and the medium was changed every 24 h. To maintain accuracy, the GCs collected in vitro did not undergo passaging. They were used directly for experiments, with fresh cells collected for subsequent experiments.

The follicular GCs were isolated and cultured. When the cell density reached 60%–70%, the cells were treated with drugs. The Erastin group was treated with 10 µmol/L Erastin (MedChemExpress, New Jersey, USA) for 48 h. In the Fer-1 group, GCs were treated with Erastin for 24 h, followed by treatment with 10 µmol/L ferrostatin-1 (Fer-1, MedChemExpress, New Jersey, USA) for another 24 h. In the Quercetin group, after 24 h of Erastin treatment, cells were treated with 10 µmol/L quercetin (MedChemExpress, New Jersey, USA) for 24 h. The dimethyl sulfoxide (DMSO) group served as the blank control group. Erastin, quercetin, and Fer-1 were all dissolved in DMSO (Solarbio, Beijing, China), with the concentration of DMSO in each group being 0.1%.

### Immunofluorescence

The slides were washed twice with PBS and fixed with 1 mL of 4% paraformaldehyde per well for 15 min. Excess paraformaldehyde was then aspirated, and the slides were slightly dried. A histochemical pen (Servicebio, Wuhan, China) was used to draw a circle around the evenly distributed cells on the coverslip to prevent antibody runoff. Then, 50–100 µL of permeabilization solution was added and incubated at room temperature for 20 min, followed by washing with PBS three times for 5 min. Within the circle, 3% BSA (Servicebio, Wuhan, China) was applied to block the tissue for 30 min at room temperature. After removing the blocking solution, the primary antibody diluted in PBS was added, and the plate was incubated overnight at 4 °C in a humid chamber. The wells were then washed three times on a decolorizing shaker (DS-2S100; Servicebio, Wuhan, China), for 5 min each. The appropriate secondary antibody was added and incubated at room temperature for 50 min, followed by another washing with PBS three times for 5 min on a decolorizing shaker. After slight drying, DAPI staining solution (Servicebio, Wuhan, China) was added within the circle and incubated in the dark at room temperature for 10 min. The slides were washed three more times with PBS (pH 7.4), each for 5 min. Finally, after drying, the slides were mounted with an anti-fading mounting medium, and images were taken using a fluorescence microscope (Nikon Eclipse C1; Nikon, Tokyo, Japan). The antibodies used are listed in Table S3.

### RNA extraction and quantitative real-time PCR

The tissue samples were grounded into powder using automatic tissue sample grinder (Shunhe, Chengdu, China), lysed by RNAiso Plus (Takara, Beijing, China) and immediately stored in −80 °C for subsequent RNA extraction. Then, 48 h after drug treatment, the RNA was extracted from the GCs using RNAiso Plus (Takara, Beijing, China). Subsequently, the TB Green^®^ Pre mix Ex Taq™ (Takara, Beijing, China) was used for the qRT-PCR analysis according to the manufacturer’s instructions, and the 2^−△△Ct^ method [21] was used to calculate the fold changes in the gene expression. The primers used for the qRT-PCR were listed in Table S2.

### Protein extraction and Western blot analysis

Phenylmethylsulfonyl fluoride (PMSF, Solarbio, Beijing, China) was diluted in a RIPA buffer (Solarbio, Beijing, China) at a 1:1,000 ratio to prepare the RIPA lysis solution. Then the tissue samples were ground into powder using an automatic tissue sample grinder (Shunhe, Chengdu, Sichuan), and lysed in the RIPA lysis buffer. GCs were seeded in six-well plates, and 48 h after drug treatment, the cell samples were collected, washed with PBS and lysed in the RIPA lysis buffer. The primary and secondary antibodies used are listed in Table S3.

### Cell counting kit-8 (CCK-8) assay

The primary GCs were cultured in a 96-well plate and in a growth medium. A cell counting kit-8 (CCK-8; MeilunBio, Dalian, China) was used for CCK-8 assay. The cells were treated with drugs, followed by the addition of 10 µL of CCK-8 reagent to each well and subsequent incubation. After 2 h of incubation, the optical density values were measured using a microplate reader (Thermo Fisher, Varioskan LUX, Waltham, USA) at 450 nm.

### Biochemical analysis in cell experiments

Antioxidant parameters [Total Glutathione (T-GSH, Elabscience, Wuhan, China); Malondialdehyde (MDA, Elabscience, Wuhan, China)] and iron levels (iron content) in the chicken GCs were measured using the biochemical kit and an iron detection kit (E-BC-K880-M, Elabscience, Wuhan, China), following the manufacturer’s instructions.

### Flow cytometry analysis

Flow cytometry was used to assess the mitochondrial membrane potential and ROS levels in the GCs. The cells were cultured in six-well plates, and then were collected 48 h post-drug treatment, washed with PBS, and lysed for 2 min with 0.25% trypsin (Gibco, Grand Island, USA). The cell suspension was centrifuged at 250 × *g* for 5 min, and the supernatant was discarded, then the cells were washed twice with PBS and centrifuged again at 250 × *g* for 5 min to obtain the cell pellet.

The JC-1 working solution was prepared by mixing 5 µL of JC-1 (500×, Beyotime, Shanghai, China) with 800 µL of sterile deionized water to dissolve and homogenize JC-1. Then, 200 µL of 5× JC-1 staining buffer was added, mixed to obtain 1 mL of JC-1 working solution. One mL of 5× JC-1 staining buffer diluted with 4 mL sterile deionized water to make 1× JC-1 staining buffer was mixed and kept on ice. Cells were resuspended in 500 µL JC-1 working solution, then 500 µL culture medium was added, and incubated at 37 °C in 5% CO_2_ for 20 min. After centrifugation at 600 × *g* for 4 min, the supernatant was discarded, and the cells were washed twice with 1× JC-1 staining buffer, 1 mL each time. Thereafter, the cells were resuspended in 500 µL 1× JC-1 staining buffer for analysis of mitochondrial membrane potential using a flow cytometer (cytoFLEX; Beckman, Brea, USA).

DCFH-DA (Beyotime, Shanghai, China) was diluted in the serum-free culture medium at a 1:1,000 ratio to a final concentration of 10 µmol/L. Each tube received 1 mL of the diluted DCFH-DA, and the cells were incubated at 37 °C for 20 min, and then they were overturned every 3 min to ensure full contact between the probe and the cells. Then the cells were centrifuged at 350 × *g* for 5 min, and then the supernatant was discarded, and the cells were washed three times with serum-free culture medium to remove any unentered DCFH-DA thoroughly. Further, the ROS levels in the cells were analyzed using FlowJo10.8.1 software (BD Biosciences, USA).

### Cell staining by fluorescent probes

The staining of ferrous ions (Fe^2+^) using a fluorescent probe was performed following the instructions provided with FerroOrange (Dojindo, Kumamoto, Japan). First, 24 µg of FerroOrange was dissolved in 35 µL of DMSO by pipetting to create a 1 mmol/L FerroOrange solution. This solution was then diluted with HBSS to prepare a 1 µmol/L FerroOrange working solution. Follicular GCs were seeded in fluorescence culture dishes (Biosharp, Hefei, China). After drug treatment, the culture medium was removed, and the cells were washed three times with HBSS. The cells were then incubated with the 1 µmol/L FerroOrange working solution at 37 °C in a 5% CO_2_ incubator for 30 min. Fluorescence was observed using a laser confocal fluorescence microscope (S3000; HOOKE Instruments Ltd., Changchun, China). Fluorescence intensity was quantified using Image J software.

### Transcriptome profiling

Cutadapt was used to remove the low-quality data, and then HISAT2v2.0.5 (Texas A&M University, Baltimore, Maryland, USA) was used to align the clean reads with the reference genes [22]. The correlation coefficient between the samples was calculated and the principal component analysis diagram was drawn. The reference genome sequence used in this study was the chicken reference genome sequence with version number GCF_000002315.6_GRCg6a in the NCBI database. Table S4 shows that the total comparison rate is approximately 90%, indicating that the data were valid. The R DESeq2 (Cornell University, Ithaca, New York, USA) was used for the identification of differentially expressed genes (DEGs) [23]. When |log_2_foldchange| ≥ 1 and *P* < 0.05 genes were considered DEGs.

### Functional enrichment analysis

ClusterProfiler (3.4.4) software was used for Gene Ontology (GO) and Kyoto Encyclopedia of Genes and Genomes (KEGG) enrichment analysis of the DEGs [24]. The *P* ≤ 0.05 was used as the threshold of difference significance.

### Statistical analysis

All the experimental data were subjected to statistical analysis using SPSS 25.0 statistical software (SPSS, Inc., Chicago, Illinois, USA). In this study, there were at least 3 biological replicates per experiment. The unpaired Student’s *t*-test was used for comparative analysis between the two groups. The experimental data were initially assessed for normal distribution, and based on this, a one-way analysis of variance (ANOVA) was conducted, including a homogeneity test of variance. One-way ANOVA, combined with Duncan’s multiple range test was used for comparative analysis of the groups, with significant level set at *P* < 0.05 (*); *P* < 0.01 (**).

## Results

### Quercetin promotes antioxidant capacity and egg production in late laying hens

**Fig. 1:**
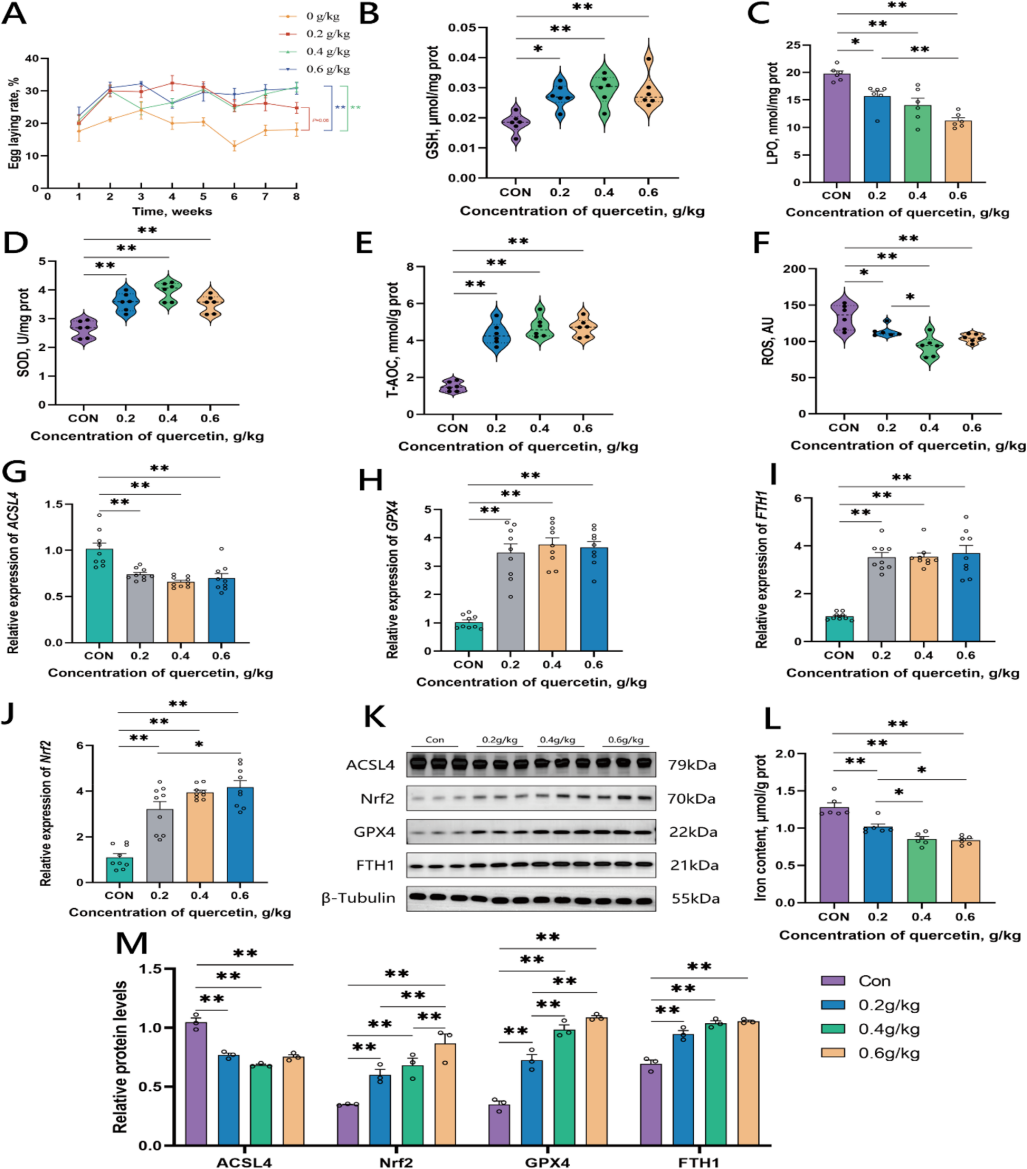
Feeding quercetin improves the laying rate in the later stages of egg production and can alleviate ferroptosis in chicken ovarian tissue. **A** The results of the laying rates of different groups fed with various concentrations of quercetin for 8 weeks. **B–F** Results of the antioxidant capacity of ovarian tissue in each group after feeding quercetin. **G**–**J** Relative expression levels of ferroptosis-related genes in the ovarian tissues of each group. **K** and **M** Relative expression levels of ferroptosis-related proteins in the ovarian tissues of each group. **L** The iron ion content in the ovarian tissues from each group. Data are presented as mean ± SEM. ^*^P < 0.05, ^**^P < 0.01

After eight weeks of feeding different concentrations of quercetin, the egg production rates of each group were analyzed (Fig. 1A). During the first week of feeding, there were not significant differences observed in the egg production rate among the experimental groups. However, from the second week, the egg-laying performance of the quercetin supplemental groups improved significantly relative to the control group. Specifically, supplementation of quercetin at 0.4 g/kg and 0.6 g/kg groups were significant, whereas, 0.2 g/kg showed some improvement as compared to the control group. This suggests that supplementation of quercetin (0.4 g/kg and 0.6 g/kg) promotes the productive performance of aging laying hens.

The results obtained in the present study indicated that feeding quercetin significantly increased the levels of GSH in the chicken ovarian tissues (Fig. 1B). The results showed that the LPO content in the 0.2 g/kg, 0.4 g/kg, and 0.6 g/kg groups was significantly lower than in the control group, with the higher LPO content observed in the 0.2 g/kg group than the 0.6 g/kg group (Fig. 1C). SOD is an important antioxidant enzyme that reduces oxidative damage to cells [25]. The results in this study showed that SOD levels in the 0.2 g/kg, 0.4 g/kg, and 0.6 g/kg groups were all significantly higher than in the control group (Fig. 1D). In addition, it was observed that T-AOC was significantly increased in the quercetin supplementation groups (0.2 g/kg, 0.4 g/kg, and 0.6 g/kg groups) as compared with the control (Fig. 1E). The results showed that the ROS content in the 0.2 g/kg, 0.4 g/kg, and 0.6 g/kg groups was reduced significantly compared to the control group, where the ROS levels in the 0.4 g/kg group was lower than the 0.2 g/kg group (Fig. 1F). Thus, it was observed in this study that quercetin enhanced the antioxidant capacity of chicken ovaries in the late egg laying period of hens.

### Quercetin alleviates ferroptosis in ovarian tissue of late-stage egg-producing chickens

We found that the relative expression level of the gene acyl-CoA synthetase long-chain family member 4 (*ACSL4*) in the 0.2 g/kg, 0.4 g/kg, and 0.6 g/kg groups were significantly downregulated compared to the control group (Fig. 1G). Furthermore, the relative expression levels of *GPX4*, ferritin heavy chain 1 (*FTH1*), and nuclear factor erythroid 2-related factor 2 (*Nrf2*) in the 0.2 g/kg, 0.4 g/kg, and 0.6 g/kg groups were significantly higher than those in the control group, with the expression levels of *Nrf2* in the 0.6 g/kg group being significantly higher than in the 0.2 g/kg group (Fig. 1H–J).

Further, Western blot analysis of the ferroptosis-related protein in ovarian tissues showed that the relative expression levels of the ACSL4 protein in the 0.2 g/kg, 0.4 g/kg, and 0.6 g/kg groups were significantly lower than those in the control group. The protein expression levels of Nrf2, GPX4, and FTH1 in the ovarian tissues of the 0.2 g/kg, 0.4 g/kg, and 0.6 g/kg groups were significantly upregulated, where the expression levels of the protein Nrf2 in the 0.6 g/kg group being significantly higher than those in the 0.2 g/kg and 0.4 g/kg groups, whereas the expression level of the GPX4 protein in the 0.2 g/kg group was lower than those in the 0.4 g/kg and 0.6 g/kg groups (Fig. 1K and M).

The occurrence of ferroptosis is closely related to abnormal iron metabolism, characterized with increased intracellular iron ion content. The iron ion content in the ovarian tissues from different experimental group was analyzed, and the results showed that the iron ion content in the ovarian tissues of the 0.2 g/kg, 0.4 g/kg, and 0.6 g/kg groups was significantly lower than that in the control group, with the iron content in the 0.2 g/kg group was higher than that in the 0.4 g/kg and 0.6 g/kg groups (Fig. 1L).

In summary, it was observed that quercetin modulates the expression of genes and proteins related to ferroptosis, and enhance the antioxidative capacity of the chicken ovarian tissues, as well as reduce the iron content in the chicken ovaries, to mitigate the occurrence of ferroptosis. Hence, quercetin concentrations at 0.4 g/kg and 0.6 g/kg significantly alleviates ferroptosis.

### Quercetin enhances the antioxidant capacity of chicken follicular GCs

**Fig. 2:**
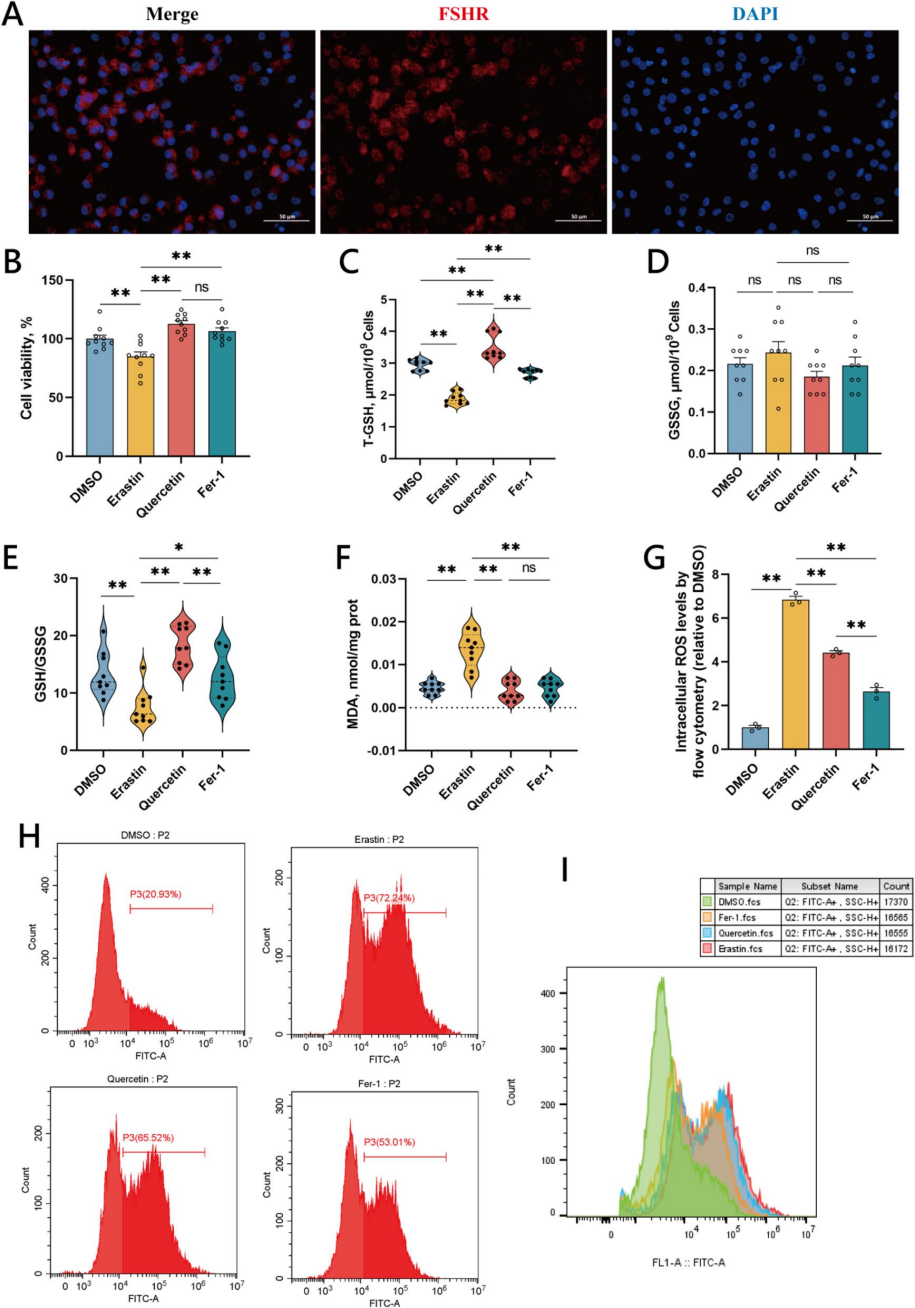
Quercetin enhances cell survival rate and antioxidant capability of chicken granulosa cells induced by Erastin. **A** Identification of chicken granulosa cells using immunofluorescence. FSHR: red, granulosa cell marker; DAPI: blue, nucleus; Merge: identification of granulosa cells. **B** After induction of cells with Erastin, cells were treated with quercetin and Fer-1, compared with the DMSO and Erastin groups, the survival rates of the different groups were detected. **C**–**F** Contents of T-GSH, GSSG, MDA, and GSH/GSSG ratio in DMSO group, Erastin group, Quercetin group, and Fer-1 group. **G** Intracellular ROS levels were measured by flow cytometry in different groups. **H** and **I** Fluorescence intensity of ROS measured by flow cytometry in partial samples. Data are presented as mean ± SEM. ^*^P < 0.05, ^**^P < 0.01, ns, not significant

The chicken GCs were collected from hierarchical follicles. Following isolation and culture, GCs were identified using FSHR immunofluorescence (Fig. 2A), as FSHR is a GC-specific protein found in the cytoplasm [26]. The morphology of the GCs observed under the microscope is presented in Fig. S3. Following quercetin treatment, the cell viability was assessed using CCK-8 (Fig. 2B). It was found that the cell viability of the GCs in the Erastin group was significantly decreased, whereas the cell viability in both the Quercetin and Fer-1 groups was significantly increased compared to the Erastin group. There was no significant difference in cell viability between the Quercetin and Fer-1 groups.

In the GCs treated with Erastin, the content of T-GSH significantly decreased relative to the control group, along with a significant reduction in the GSH/Glutathione disulfide (GSH/GSSG) ratio. The Quercetin and Fer-1 groups showed significant increase in both T-GSH content and GSH/GSSG ratio compared to the Erastin group; notably, T-GSH content and GSH/GSSG ratio in the Quercetin group were significantly higher than those in the Fer-1 group. There was no significant difference in the GSSG content among the treatment groups. This indicates that oxidative stress occurs in GCs following Erastin treatment, with both Erastin and Fer-1 capable of upregulating the cellular T-GSH content and the GSH/GSSG ratio, thereby reducing the level of oxidative stress within cells (Fig. 2C–E).

Figure 2F showed a significant increase in the MDA content in the Erastin group, whereas a significant decrease was observed in the Quercetin and Fer-1 groups, and then the treatment with quercetin and Fer-1 reduced the MDA levels in the cells. The accumulation of ROS inside cells can lead to cell damage and excessive ROS can cause oxidative stress within cells. Using flow cytometry to detect ROS (Fig. 2G–I), it was observed that the ROS level in the Erastin group significantly increased as compared with the control.

Conversely, ROS levels in both the Quercetin and Fer-1 groups were significantly lower than in the Erastin group, with the Fer-1 group displaying significantly lower ROS levels than the Quercetin group, indicating the lowest ROS levels within the Fer-1 treated cells. Both quercetin and Fer-1 treatments were effective in reducing ROS levels within cells.

Thus, it was observed that Erastin induces oxidative stress in cells and also decrease cellular antioxidant capacity. After treatment with quercetin or Fer-1, the level of oxidative stress within cells is reduced, and the cellular antioxidant capacity is increased. Therefore, quercetin can enhance the antioxidant capacity of the GCs as well as reduced the level of oxidative stress in the cells.

### Quercetin inhibits ferroptosis in GCs

**Fig. 3:**
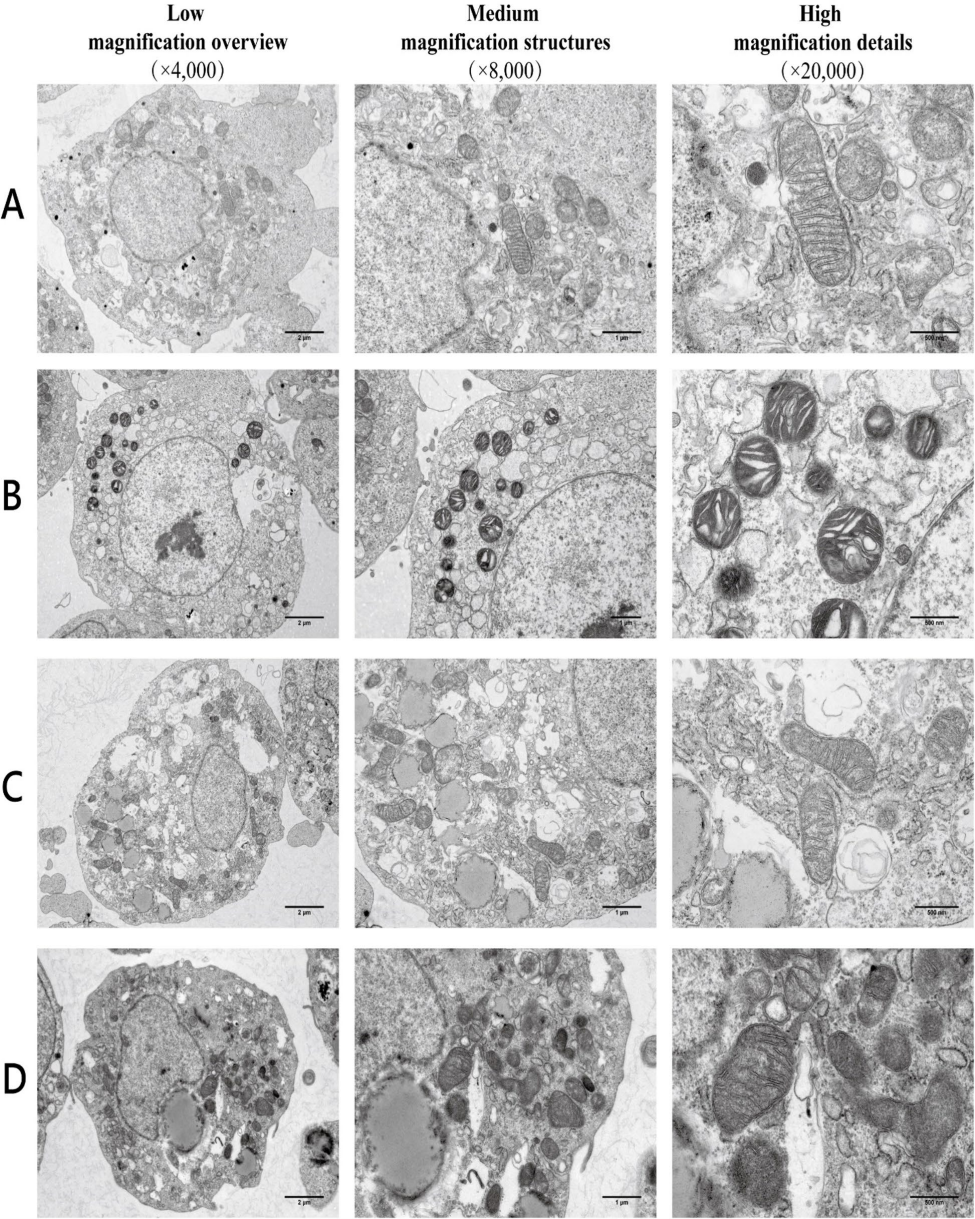
Morphology of the granule cells and mitochondrial. Representative images of granule cells and mitochondrial morphology observed via transmission electron microscopy, with magnifications scaling from smaller to larger, and scale bars corresponding to 2 μm, 1 μm, and 500 nm, respectively. **A** DMSO group. **B** Erastin group. **C** Quercetin group. **D** Fer-1 (Ferrostatin-1) group

Transmission electron microscopy observation of cellular morphology (Fig. 3) reveals that in the DMSO group, cells display normal morphology, with intact mitochondrial double membrane structures and clear cristae. In the Erastin group, the cells showed ruptured mitochondrial outer membranes and reduced or absent cristae. In the Quercetin and Fer-1 groups, it was observed that the mitochondrial outer membrane integrity is restored, and the number of cristae increases. This indicates that quercetin and Fer-1 possess functions akin to ferroptosis inhibitors, capable of alleviating morphological characteristics of ferroptosis induced by Erastin, such as mitochondrial membrane rupture and reduction in cristae.

**Fig. 4:**
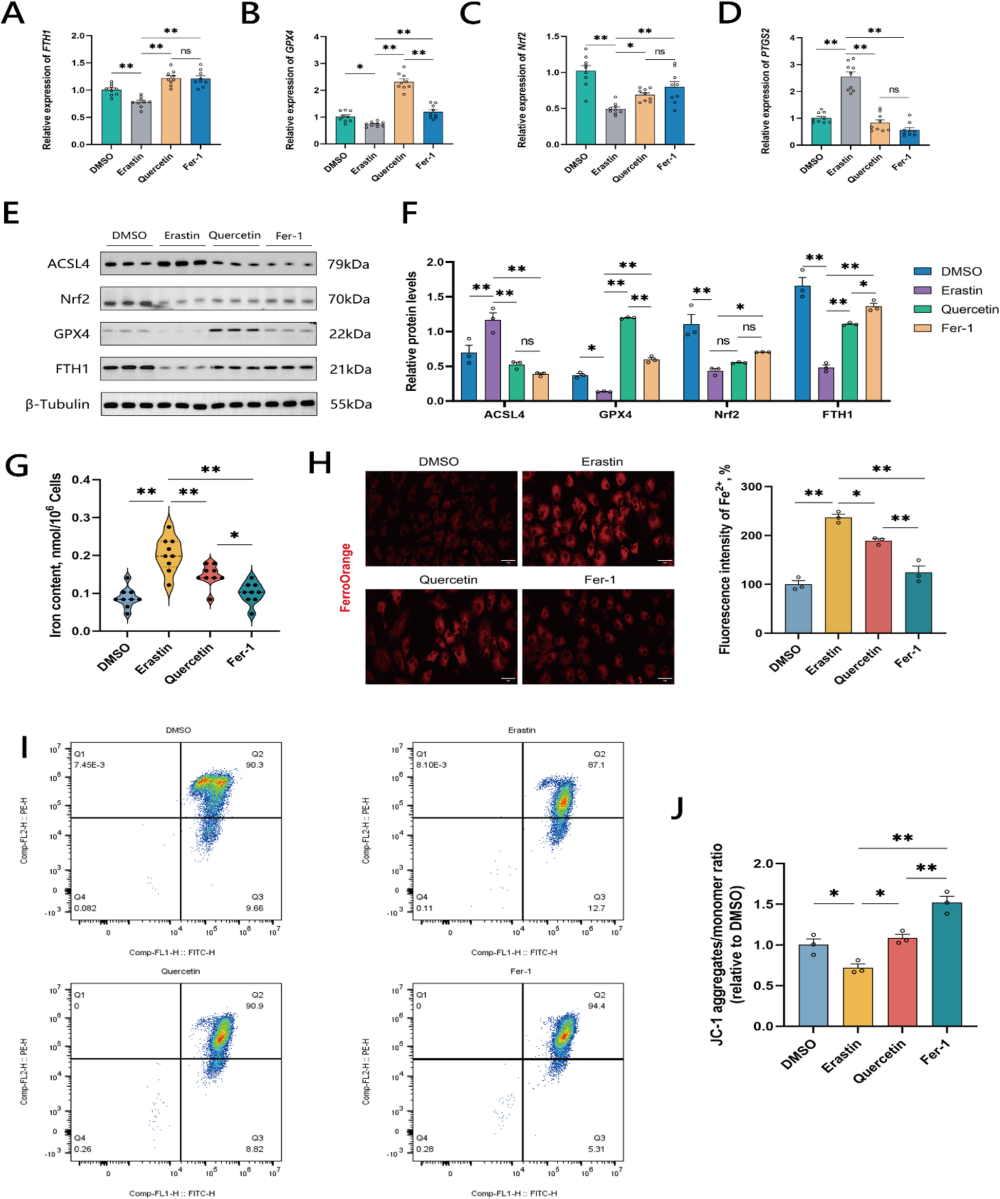
Quercetin significantly inhibits ferroptosis in chicken granule cells. **A–D** RT-qPCR results of ferroptosis-related genes *FTH1*, *GPX4*, *Nrf2*, *PTGS2* in chicken granule cells. **E** and **F** Western blot results for ferroptosis-related proteins ACSL4, Nrf2, GPX4, FTH1 in chicken granule cells. **G** Measurement of intracellular iron ion content in different groups of chicken granule cells. **H** Fluorescence imaging of Fe^2+^ in granulocytes using the FerroOrange probe. The left side displays representative confocal images for each group of cells, and the right side bar graph shows the fluorescence intensity of Fe^2+^ within granulocytes. **I** Representative results of mitochondrial membrane potential in DMSO, Erastin, Quercetin, and Fer-1 groups using flow cytometry JC-1 assay. **J** Results of mitochondrial membrane potential in granule cells measured by flow cytometry JC-1 assay. Data are presented as mean ± SEM. ^*^P < 0.05, ^**^P < 0.01, ns, not significant

Further, the expression levels of the genes related to ferroptosis were measured. Thus, in the Erastin group, the expression levels of *FTH1*, *GPX4*, and *Nrf2* were significantly lower than in the DMSO group. In contrast, expression levels of *FTH1*, *GPX4*, and *Nrf2* in the Quercetin and Fer-1 groups were significantly increased compared to the Erastin group; notably, *GPX4* gene expression in the Quercetin group was significantly higher than in the Fer-1 group (Fig. 4A–C). *PTGS2* (Prostaglandin-endoperoxide synthase 2), a key regulatory gene involved in inflammatory responses, cell proliferation, and differentiation, and serving as a biomarker for ferroptosis without driving the process itself, was significantly upregulated in the granulocytes after Erastin induction. However, *PTSG2* expression in the Quercetin and Fer-1 groups was significantly lower than in the Erastin group (Fig. 4D).

Western blot analysis of the expression levels of proteins related to ferroptosis showed that the expression levels of GPX4, Nrf2, and FTH1 proteins were significantly lower in the Erastin group compared to the DMSO group, while the expression of ACSL4 was significantly higher in the Erastin group. In the Quercetin and Fer-1 groups, the expression levels of GPX4 and FTH1 were significantly higher than in the Erastin group, and the expression levels of ACSL4 were significantly lower than in the Erastin group. The expression level of Nrf2 was significantly higher in the Fer-1 group compared to the Erastin group. The expression of GPX4 was significantly higher in the Quercetin group compared to the Fer-1 group, and the expression of FTH1 was significantly lower in the Quercetin group compared to the Fer-1 group (Fig. 4E and F), which is in agreement with the RT-qPCR results obtained in this study. The results indicate that treatment with quercetin and Fer-1 in granulocytes can inhibit ferroptosis within the cells, and the higher expression level of GPX4 in the quercetin-treated cells may reveal certain superior antioxidant capabilities in the Quercetin group compared to the Fer-1 group.

Excessive accumulation of iron in cells is a hallmark event in the process of ferroptosis. Upon assessing cellular iron content, we observed a significant increase in iron levels in the Erastin group compared to the DMSO group. Conversely, iron levels in both the Quercetin and Fer-1 groups were significantly reduced compared to the Erastin group, with the Fer-1 group displaying a notably lower iron content than the Quercetin group. This suggests that both quercetin and Fer-1 are capable of reducing intracellular iron levels, with Fer-1 exerting significant effect (Fig. 4G). Furthermore, using confocal microscopy to observe GCs stained with the FerroOrange fluorescent probe, we found that ferrous ion levels mirrored the total iron levels. The Erastin group showed significantly higher ferrous ion levels compared to the DMSO group. In contrast, both the Quercetin and Fer-1 groups had significantly lower ferrous ion levels compared to the Erastin group, with the Fer-1 group exhibiting even lower levels than the Quercetin group (Fig. 4H). This indicates that both quercetin and Fer-1 can reduce intracellular iron and ferrous ion levels, with Fer-1 exerting significant effect.

During the process of ferroptosis, a decrease in mitochondrial membrane potential leads to mitochondrial dysfunction, preventing cells from maintaining normal energy metabolism. Mitochondrial membrane potential was measured using flow cytometry. Cells in the Erastin group showed a significant decrease in the mitochondrial membrane potential, whereas cells in the Quercetin group exhibited a significantly higher mitochondrial membrane potential compared to the Erastin group. The mitochondrial membrane potential in cells of the Fer-1 group was significantly higher compared to both the Erastin and Quercetin groups (Fig. 4J). The flow cytometry scatter plots also reflected this trend (Fig. 4I).

In summary, determining factors related to ferroptosis and considering the effects of quercetin on the antioxidant capabilities of granulocytes showed that quercetin alleviates ferroptosis in chicken granulocytes.

### Transcriptome sequencing and DEGs analysis

The transcriptome sequencing of the three experimental groups (DMSO, Erastin, and Quercetin) of 9 samples in total, yielded 62.75 G of Clean Data. The effective data volume for each sample ranged between 6.92 and 7.02 G, with a Q30 base distribution of 94.05% to 96.13% and an average GC content of 48.03%. Mapping the reads to the reference genome revealed the genome alignment for each sample, with alignment rates between 93.93% and 95.36%. Based on these alignments, an analysis of the expression levels of protein-coding genes was conducted.

**Fig. 5:**
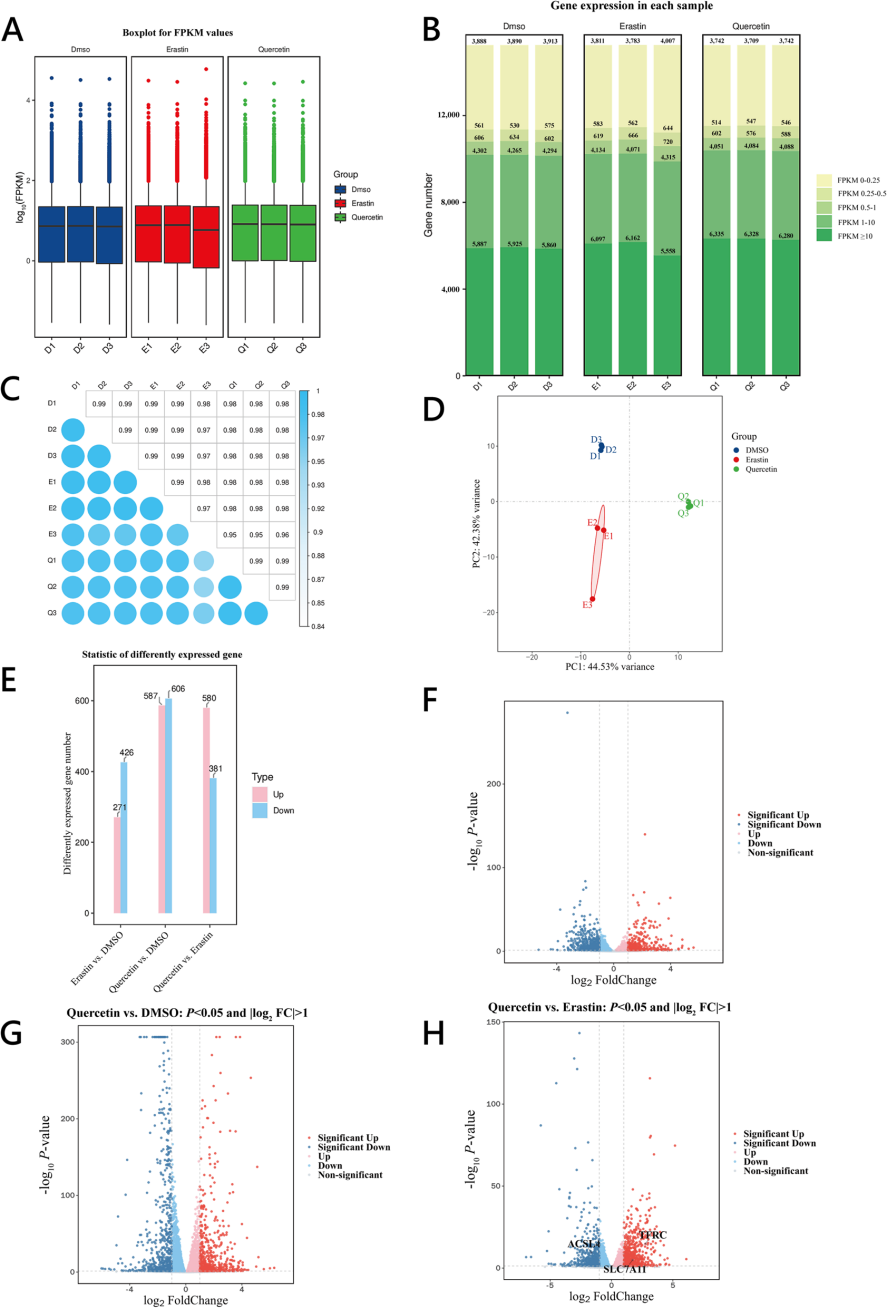
Granulocyte transcriptome sequencing overview analysis. **A** Boxplot of gene FPKM values for each sample, with the *x*-axis representing sample names and the *y*-axis representing log_10_(FPKM). Each boxplot corresponds to five statistical measures (from top to bottom: maximum, third quartile, median, first quartile, and minimum). **B** Distribution chart of FPKM expression levels, with different colors indicating different ranges of FPKM values, the *x*-axis represents samples, and the *y*-axis represents the number of protein-coding genes. **C** Heatmap of the correlation coefficients between samples, with sample names as both axes and colors indicating the magnitude of the correlation coefficient. **D** PCA analysis result plot for the samples. **E** Statistical chart of differentially expressed gene counts from the transcriptome sequencing. **F**–**H** Volcano plots of differentially expressed genes, with the *x*-axis representing log_2_ (Fold Change) and the *y*-axis representing −log_10_*P* value. The dashed line indicates the threshold line for differential gene screening criteria

Using the Fragments Per Kilobase of transcript per Million mapped reads (FPKM) to account for the sequencing depth and gene length, a threshold for the gene expression was established at FPKM > 1, indicating gene expression when FPKM values exceeded this threshold (Fig. 5A).

Given the variability in the number and distribution of gene expression levels across samples, gene expression levels (FPKM) were categorized into different intervals. The number of genes expressed within these intervals was calculated and visually represented in a stacked bar chart (Fig. 5B).

Further, correlation coefficients were computed from the FPKM values to assess sample similarity, resulting in a heatmap of correlation coefficients (Fig. 5C). Each sample pair exhibited a correlation coefficient greater than 0.8, indicating good reproducibility between the replicates and suitability for further analysis. Principal Component Analysis (PCA) was conducted (Fig. 5D) to verify sample dispersion across different groups and clustering within the same group, confirming the reliability of the sequencing results and paving the way for differential gene analysis.

Bioinformatics analysis of the cells from different groups (Fig. 5E–H) revealed differential gene expression. Between the DMSO and Erastin groups, 697 DEGs were identified, with 271 upregulated and 426 downregulated. The comparison between the Quercetin and DMSO groups unveiled 1,193 DEGs, with 587 upregulated and 606 downregulated. Lastly, comparing the Quercetin and Erastin groups revealed 961 DEGs, with 580 upregulated and 381 downregulated.

### GO and KEGG pathway enrichment analysis

**Fig. 6:**
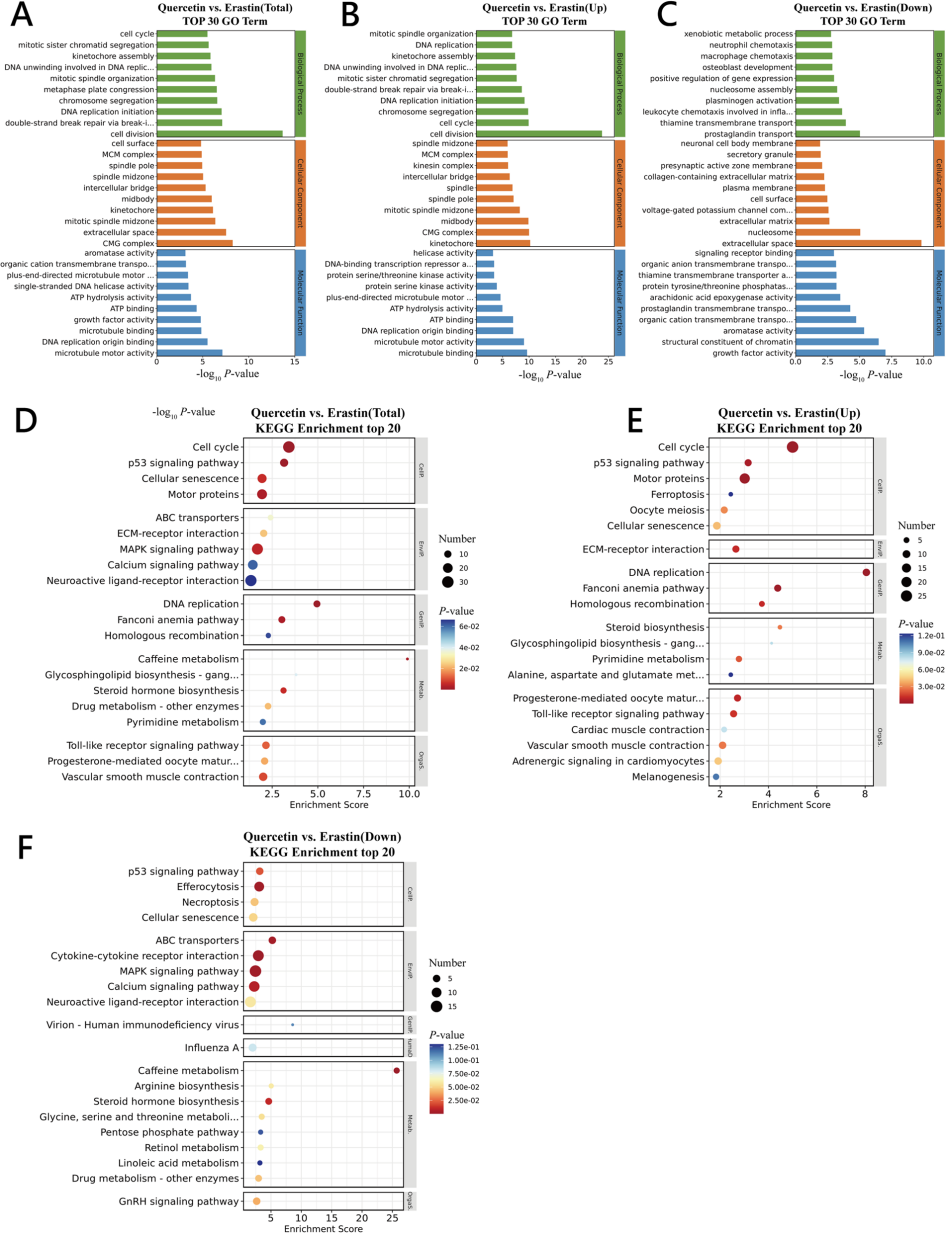
Results of GO and KEGG enrichment analysis for Quercetin and Erastin groups. **A**–**C** Top pathways in GO enrichment analysis of differentially expressed genes in Quercetin and Erastin groups. The *y*-axis represents GO terms, and the *x*-axis represents the significance level of GO term enrichment. **D**–**F** Top pathways in KEGG enrichment analysis of differentially expressed genes in Quercetin and Erastin groups. The *x*-axis represents the enrichment score, the *y*-axis represents KEGG terms, bubble color indicates significance, and bubble size reflects the number of genes enriched in the pathway

In this study the GO and KEGG enrichment analyses of the DEGs between the Quercetin group and the Erastin group cells was conducted (additional result: Fig. S4 and S5). The GO enrichment analysis of both the Quercetin and Erastin groups showed that the 10 most significant terms from the categories of Biological Process (BP), Cellular Component (CC), and Molecular Function (MF) were selected for visualization (Fig. 6A–C).

In the BP category, pathways enriched in both groups were primarily involved in crucial biological processes such as cell cycle regulation, DNA replication and repair, chromosome segregation, and division. This suggests that quercetin and Erastin treatments might affect follicular GCs proliferation, differentiation, or apoptosis by impacting these fundamental cellular biology processes. Difference in the CC category was mainly enriched in the aspects related to cell cycle regulation, cell division, and communication between cells and their external environment. Specifically, enrichment at the cell surface may be associated with interactions between cells and their external environment, such as receptor activation and cell adhesion. In the MF context, the TOP pathway is predominantly enriched in a wide range of molecular functions vital for normal cell growth, division, and metabolism.

Thus, the effects of quercetin and Erastin on chicken ovarian follicle GCs involve a series of complex biological processes, including the regulation of the cell cycle, DNA replication and repair, and regulatory hormones.

Through the KEGG enrichment analysis of DEGs between the Erastin-treated cells and quercetin-treated cells (Fig. 6D–F), transcriptomic sequencing analysis of the ovarian follicular GCs revealed significant differences in multiple key biological pathways between the Erastin-treated chicken GCs and normal chicken GCs, particularly in antioxidant, apoptosis, ferroptosis, and inflammation-related pathways.

Thus, Quercetin treatment group exhibited significant alterations in cell apoptosis-related pathways. Notably, within the p53 signaling pathway. The p53 protein is a key regulatory factor in cell cycle control and apoptosis, indicating that quercetin may influence cell apoptosis mechanisms through the modulation of p53 signaling pathway. Further, the ferroptosis pathway was significantly downregulated in the Quercetin-treated group, where *ACSL4*, *SLC7A11* (Solute carrier family 7 member 11), and *TFRC* (Transferrin receptor) were selected as the potential target genes responsible for the mitigation role of quercetin on iron death in chicken GCs. Inflammation-related pathways, such as the Toll-like receptor signaling pathway, were downregulated in the Quercetin-treated group, suggesting that quercetin affects inflammation responses.

In the differential expression gene KEGG enrichment analysis of the Erastin group and Quercetin group, potential target genes *ACSL4*, *SLC7A11*, and *TFRC* were selected for validation through real-time quantitative fluorescence. The results, as shown in Fig. S6, demonstrate that the real-time fluorescence quantitative results are consistent with the sequencing results, indicating reliable sequencing data. *ACSL4*, *SLC7A11*, and *TFRC* are potential target genes for quercetin in alleviating ferroptosis in granular cells.

### Quercetin alleviates apoptosis in chicken GCs

**Fig. 7:**
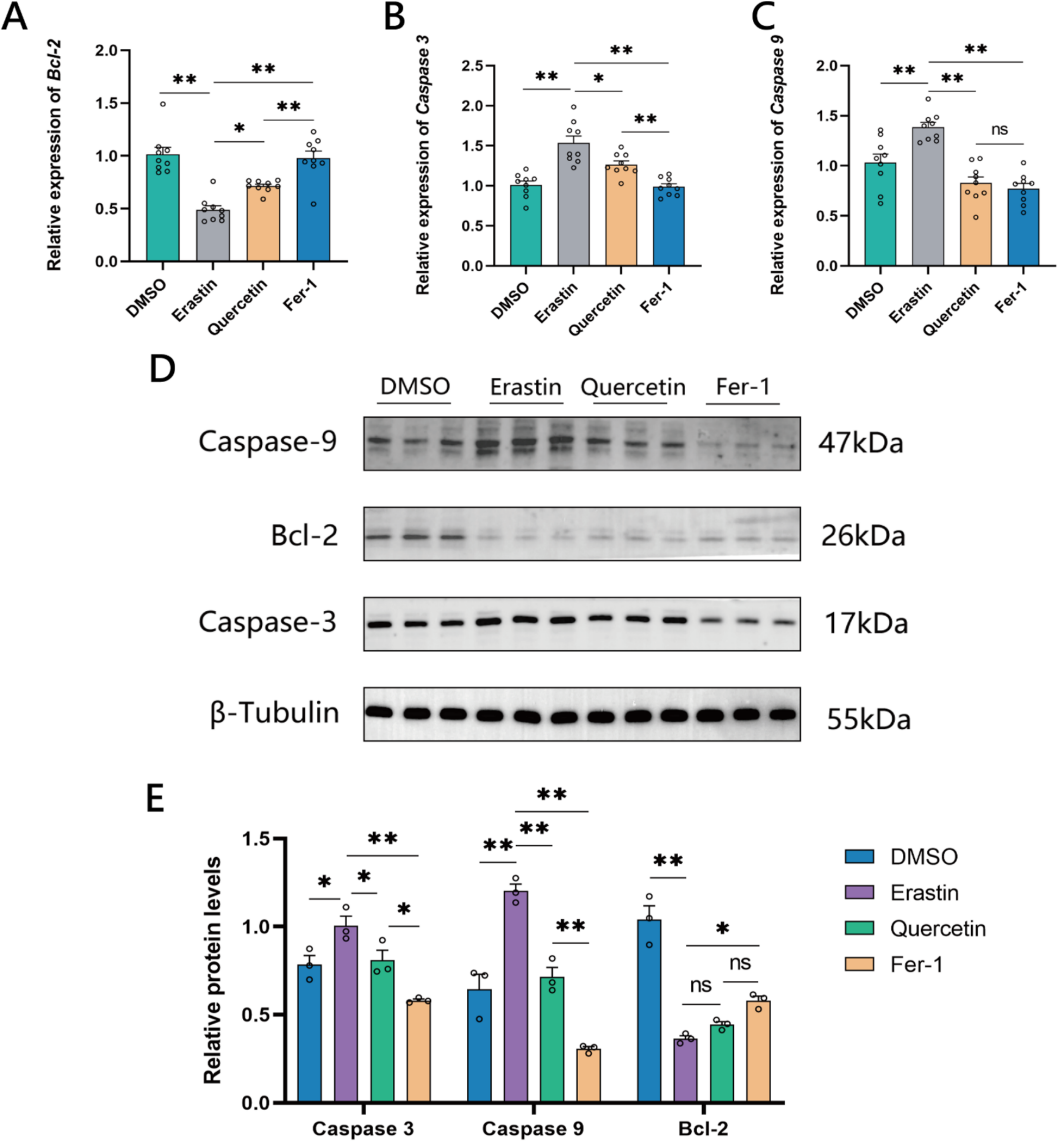
Quercetin alleviates apoptosis in chicken granulocytes. **A**–**C** RT-qPCR results of apoptosis-related genes *Bcl-2*, *Caspase 3*, and *Caspase 9* in chicken granulocytes. **D** and **E** Western Blot results of apoptosis-related genes Bcl-2, Caspase 3, and Caspase 9 in chicken granulocytes. *P < 0.05, **P < 0.01, ns, not significant

RT-qPCR analysis showed expression levels of apoptosis-related genes *Bcl-2* (B-cell lymphoma 2), *Caspase 3* (Cysteine-aspartic protease 3), and *Caspase 9* (Cysteine-aspartic protease 9) (Fig. 7A–C). The Erastin group showed significantly higher expression levels of *Caspase 3* and *Caspase 9* compared to the DMSO group, whereas the expression of *Bcl-2* significantly lower. Also, both the Quercetin and Fer-1 groups exhibited significantly higher expression of *Bcl-2* compared with the Erastin group, and significantly lower expressions of *Caspase 3* and *Caspase 9*. Quercetin group showed significantly lower *Bcl-2* expression and significantly higher Caspase 3 expression compared to the Fer-1 group.

The results in Fig. 7D and E showed that the expression levels of Caspase-3 and Caspase-9 proteins in the Erastin group were significantly higher than those in the DMSO group, whereas, the expression level of Bcl-2 in the Erastin group was significantly lower than that in the DMSO group. Both Quercetin and Fer-1 groups showed significantly lower expressions of Caspase 3 and Caspase 9 proteins compared to the Erastin group, with the Fer-1 group having significantly higher Bcl-2 expression than the Erastin group.

The expression of the protein Caspase-3 was significantly higher in the Quercetin group than that of the Fer-1 group, and the expression of the protein Caspase-9 was also significantly higher in the Quercetin group compared to the Fer-1 group, with no significant difference in the expression Bcl-2 protein between the two groups. Following Erastin treatment, apoptosis occurred in granulocytes, and both quercetin and Fer-1 could alleviate apoptosis in granulocytes caused by Erastin, with Fer-1 showing a superior inhibitory effect on granulocyte apoptosis compared to quercetin.

### Quercetin alleviates inflammation in chicken GCs

**Fig. 8:**
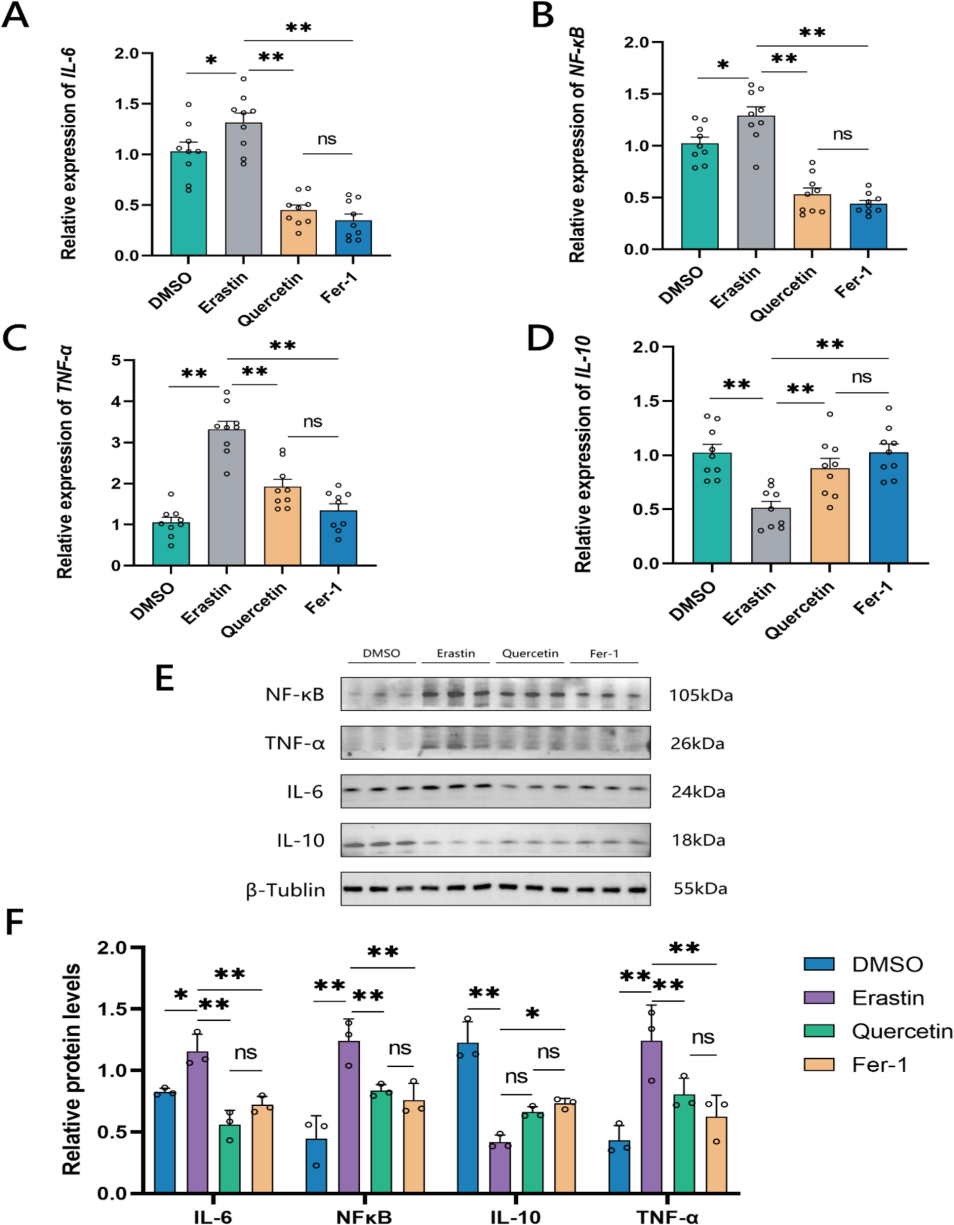
Quercetin alleviates inflammation in chicken granulocytes. **A**–**C** RT-qPCR results of inflammation-related genes *IL-6*, *NF-κB*, *TNF-α*, and *IL-10* in chicken granulocytes. **E** and **F** Western Blot results of inflammation-related genes IL-6, NF-κB, TNF-α, and IL-10 in chicken granulocytes. *P < 0.05, **P < 0.01, ns, not significant

RT-qPCR analysis revealed the expression levels of inflammation-related genes such as Interleukin-6 (*IL-6*), Nuclear factor kappa-light-chain-enhancer of activated B cells (*NF-κB*), Tumor necrosis factor-alpha (*TNF-α*), and Interleukin-10 (*IL-10*) (Fig. 8A–D). The Erastin group exhibited significantly higher expression of *IL-6*, *NF-κB*, and *TNF-α* compared to the DMSO group, whereas *IL-10* showed lower expression. Further, both Quercetin and Fer-1 groups showed significantly lower expression levels of *IL-6*, *NF-κB*, and *TNF-α* compared to the Erastin group, and significantly higher expression of *IL-10*. No significant difference was observed in the expression of inflammation-related genes between the Quercetin and Fer-1 groups.

Figure 8E and F showed that the expression of Western blot analysis of the inflammatory related proteins such as NF-κB, TNF-α, IL-6 and IL-10 in treatment groups. Following the induction with Erastin, the expression levels of IL-6, NF-κB, and TNF-α proteins were significantly upregulated, while the expression level of IL-10 was significantly downregulated, indicating an inflammatory response in the granulocytes. Further, quercetin and Fer-1 significantly lowered the expression of IL-6, NF-κB, and TNF-α proteins compared to the Erastin group. It was further observed that Fer-1 regulated high expression of IL-10 protein than the Erastin group with no significant difference in the protein expression of IL-10 between the Quercetin and the Erastin groups.

These results indicated that Erastin treatment of granulocytes can induce inflammatory response, and treatment with either quercetin or Fer-1 can alleviate this inflammatory response in the granulocytes, with no significant difference between the two treatments.

## Discussion

Ferroptosis is an iron-dependent form of cell death characterized by the accumulation of lipid peroxides, ultimately leading to cell death. While its role has been extensively studied in human and animal systems, it is a relatively new research focus in livestock, particularly poultry [27–29]. The aim of this study was to investigate the impact of quercetin on ferroptosis in chicken GCs and identify the underlying molecular mechanisms involved. The results from our transcriptome sequencing revealed that quercetin targets *ACSL4*, a gene known to play a crucial role in ferroptosis by promoting lipid peroxidation. Consistent with findings in porcine ovarian GCs, where *ACSL4* was identified as part of the *Akt/FHC/ACSL4* signaling pathway in regulating ferroptosis [30]. We found that quercetin significantly reduced ferroptosis in chicken GCs by modulating this pathway. Similarly, previous studies in ovine GCs identified miR-134-3p and *HMOX1* as regulators of ferroptosis [31], further supporting the role of ferroptosis in ovarian health across species. Hence, the results from this present study adds to this growing body of research by providing the first evidence of the protective role of quercetin against ferroptosis in the ovaries of aged laying hens.

Ferroptosis and apoptosis are distinct types of cell death, each with unique molecular mechanisms, morphological features, and physiological roles. For example, previous studies have shown that *FBW7* can induce both ferroptosis and apoptosis in pancreatic cancer cells [32], and miR-93-5p plays a role in regulating both processes in GCs of polycystic ovary syndrome patients [33]. While ferroptosis and apoptosis represent separate pathways of cell death, their interaction and mutual influence in animal health and disease cannot be overlooked [32, 34, 35]. Our study similarly found that Erastin, a ferroptosis inducer, also triggered apoptosis in chicken GCs, while Fer-1, a ferroptosis inhibitor, reduced this apoptotic response. This suggests a potential crosstalk between ferroptosis and apoptosis in chicken GCs, which may have broader implications for understanding ovarian function in poultry.

The ability of quercetin to mitigate ferroptosis aligns with its well-documented antioxidant properties [36], alleviating oxidative stress [37], thwarting chronic diseases [38], and safeguarding cellular well-being [15]. In our previous study, we demonstrated that quercetin enhances antioxidant enzyme activities in aged roosters [39], which correlates with our current finding that quercetin increases antioxidant capacity in the ovaries of aged laying hens. This is consistent with other studies which shows that quercetin alleviates oxidative stress and inflammation by regulating key pathways such as NOX2/ROS/NF-κB [40, 41]. Additionally, quercetin has been discovered to decrease the production of inflammatory factors, cell proliferation, and *NF-κB* activation in LPS-induced microglial cells [41]. Other studies showed that quercetin effectively regulates energy deficits and inflammatory responses in a chicken embryo inflammation model induced by lipopolysaccharide [42]. In this present study, we treated GCs induced by Erastin with quercetin and measured the expression levels of *NF-κB*, *TNF-α*, *IL-6*, and other inflammatory factors, and the results indicated that quercetin could modulate the expression of inflammatory factors in GCs, consequently suppressing the inflammatory response, which aligns with studies discussed earlier. Studies indicate that quercetin can hinder apoptosis in chondrocytes by obstructing the MAPK signaling pathway [43]. Moreover, quercetin has been shown to diminish the viability of prostate cancer cells by prompting apoptosis and necrosis, impacting mitochondrial integrity, and disrupting ROS balance, while leaving normal prostate epithelial cells unaffected [44]. As a potential anticancer agent in cancer therapy, quercetin exhibits antioxidant, anti-inflammatory, and anti-apoptotic properties, along with the capability to inhibit various forms of programmed cell death. It can be employed alone or in conjunction with established chemotherapy and radiotherapy protocols to bolster therapeutic effectiveness and minimize side effects [45, 46]. Additionally, the anti-inflammatory and antioxidant attributes of quercetin present therapeutic promise for other conditions such as cardiovascular diseases [47] and neurodegenerative diseases [48, 49]. Our results support the hypothesis that quercetin exerts its anti-ferroptotic effects by modulating inflammatory and oxidative stress pathways in GCs.

The novelty of our study lies in the discovery of the inhibitory effect of quercetin on ferroptosis in chicken GCs, mediated through key genes such as *ACSL4*, *SLC7A11*, and *TFRC*. However, other plant polyphenols including resveratrol, catechins, and curcumin have been shown to regulate ferroptosis in various cell types [50–52]. Our study is one of the first to investigate the role of quercetin to mitigates ferroptosis in poultry. The results from this study expands our understanding of ferroptosis in non-mammalian species as well as revealed that quercetin could serve as a dietary supplement to improve ovarian health and productivity in poultry by combating inflammation, oxidative stress, and regulating apoptosis and ferroptosis, thereby offering insights for future cutting-edge studies on quercetin.

Numerous studies have reported that *ACSL4* is a crucial regulator of ferroptosis [53], catalyzing the acylation of polyunsaturated fatty acids [28], promoting lipid peroxidation, and triggering ferroptosis. *TFRC* regulates intracellular iron levels by mediating cellular iron uptake [54, 55]. *SLC7A11* was reported to regulate the antiporter activity for glutamine and cystine, promotes the synthesis of GSH, enhances cellular antioxidant capacity, and inhibits ferroptosis. In our study, we found that quercetin significantly altered the expression of *Nrf2*, *SLC7A11*, and *GPX4*, improving ferroptosis in chicken ovarian GCs. This is consistent with a study that reported that kaempferol mitigates neuronal ferroptosis by activating the Nrf2/SLC7A11/GPX4 axis [56]. In summary, our study demonstrates that quercetin inhibits ferroptosis in chicken GCs by targeting key genes such as *ACSL4*, *SLC7A11*, and *TFRC*. This improves ovarian health in aged laying hens as well as highlights the potential for quercetin as a dietary supplement in the poultry industry. Further cutting-edge research is needed to explore the broader applications of quercetin and other polyphenols in regulating ferroptosis and enhancing animal health. According to the results obtained in this present study, we hypothesize that quercetin mitigates ferroptosis in chicken GCs by targeting the *ACSL4/SLC7A11/TFRC* axis, thereby enhancing antioxidant defense mechanisms and alleviating lipid peroxidation. These results suggest that quercetin could be utilized as a therapeutic agent in poultry farming to enhance ovarian health, improve egg production, and extend the reproductive lifespan of laying hens. Furthermore, given quercetin’s ability to modulate multiple forms of cell death, it holds promise for applications in cancer therapy [45, 46], as well as in the treatment of inflammatory and neurodegenerative diseases [47–49]. In this present study, we identified key genes involved in quercetin-mediated ferroptosis inhibition, future studies should explore the broader signaling networks influenced by quercetin in GCs. Furthermore, the application of quercetin in poultry production should be further investigated, including its long-term effects on reproductive performance and overall health.

## Conclusion

Taken together, this study revealed that quercetin supplementation improves ovarian function in chickens during the late egg-laying period, while reducing ovarian oxidative stress and mitigating the effects of ferroptosis on the ovary. Antioxidant analysis suggest that quercetin enhances the antioxidative capacity of chicken GCs. In addition, quercetin reduces intracellular iron and ROS levels, restores mitochondrial membrane potential disrupted by Erastin, and inhibits Erastin-induced ferroptosis. Transcriptome sequencing revealed that Erastin induces apoptosis and inflammation in chicken ovarian follicle GCs. By analyzing gene and protein expression levels of apoptosis-related markers (*Caspase-3*, *Caspase-9*, and *Bcl-2*) and inflammatory factors (*NF-κB*, *TNF-α*, *IL-6*, and *IL-10*), we found that quercetin alleviates Erastin-induced apoptosis and inflammation. Key genes involved in ferroptosis (*ACSL4*,* SLC7A11*, and *TFRC*) were identified following quercetin treatment. This present study suggest that quercetin protects poultry ovarian tissues from ferroptosis damage, thereby providing a theoretical basis for its application in hens during the late laying period and highlights the potential molecular mechanisms through which quercetin mitigates ferroptosis in chicken follicle GCs.

## Supplementary Information

Additional file 1: Fig. S1. Transmission electron micrographs of the atretic (A) and normal (B) follicles. The white triangles represented the lipid droplets; the black arrows showed mitochondria with structural defects; the white arrows showed the mitochondria with intact structures; the white dashed boxes delineate areas with a large accumulation of lipid droplets. Fig. S2 Iron levels of the atretic and normal follicles. Data are presented as mean ± SEM. ^*^P < 0.05, ^**^P< 0.01. Fig. S3 Morphology of granulosa cells under the electronic microscope. Fig. S4 GO and KEGG enrichment analysis of the differentially expressed genes between the erastin and DMSO groups. A–C Top pathways in GO enrichment analysis of the differentially expressed genes in the Erastin and DMSO groups. The *y*-axis represents GO terms, and the *x*-axis represents the significance level of the GO enrichment terms. D–F Top pathways in KEGG enrichment analysis of the differentially expressed genes in the Erastin group and DMSO groups. The *x*-axis showed the enrichment score, the *y*-axis showed the KEGG terms, bubble color indicates significance, and bubble size reflects the number of genes enriched in the pathway. Fig. S5 GO and KEGG enrichment analysis of the differentially expressed genes between the quercetin and DMSO groups. A–C Top pathways in GO enrichment analysis of the differentially expressed genes in Quercetin and DMSO groups. The *y*-axis represents the GO terms, and the *x*-axis represents the significance level of the GO enrichment terms. D–F Top pathways in KEGG enrichment analysis of the differentially expressed genes in Quercetin and DMSO groups. The *x*-axis represents the enrichment score, the *y*-axis represents KEGG terms, bubble color indicates significance, and bubble size reflects the number of genes enriched in the pathway. Fig. S6 RT-qPCR validation results of target genes filtered from sequencing data. Validation results of the target genes (*ACSL4*, *SLC7A11*, and *TFRC*) filtered from transcriptome sequencing data were obtained via RT-qPCR. The RT-qPCR results were consistent with the transcriptome sequencing results. Table S1 The composition and nutritional values of the basal diet (%, dry matter). Table S2 Primers used for the quantitative real-time PCR (qRT-PCR). Table S3 Antibodies used in this study. Table S4 Comparison of the adipose sequencing data with the reference genome. Table S5 Potential target genes affecting ferroptosis among differentially expressed genes between quercetin and erastin.

## Data Availability

The datasets presented in this study can be found in online repositories. The names of the repository/repositories and accession number(s) can be found at: https://www.ncbi.nlm.nih.gov/bioproject/PRJNA1128667.

## Abbreviations

ACSL4: Acyl-CoA synthetase long-chain family member 4
Bcl-2: B-cell lymphoma 2
BP: Biological process
Caspase 3: Cysteine-aspartic protease 3
Caspase 9: Cysteine-aspartic protease 9
CC: Cellular component
CCK-8: Cell counting kit-8
DEG: Differentially expressed gene
DMSO: Dimethyl sulfoxide
Fer-1: Ferrostatin-1
FPKM: Fragments per kilobase of transcript per million mapped reads
FTH1: Ferritin heavy chain 1
GC: Granulosa cell
GO: Gene ontology
GPX4: Glutathione peroxidase 4
GSH: Glutathione
GSSG: Glutathione disulfide
HBSS: Hank’s balanced salt solution
IL-10: Interleukin-10
IL-6: Interleukin-6
KEGG: Kyoto Encyclopedia of Genes and Genomes
LPO: Lipid peroxidation
MDA: Malondialdehyde
MF: Molecular function
NF-κB: Nuclear factor kappa-light-chain-enhancer of activated B
Nrf2: Nuclear factor erythroid 2-related factor 2
PBS: Phosphate buffered saline
PCA: Principal components analysis
PMSF: Phenylmethylsulfonyl fluoride
PTGS2: Prostaglandin-endoperoxide synthase 2
ROS: Reactive oxygen species
SLC7A11: Solute carrier family 7 member 11
SOD: Superoxide dismutase
T-AOC: Total antioxidant capacity
TFRC: Transferrin receptor
T-GSH: Total glutathione
TNF-α: Tumor necrosis factor-alpha
