# Supplementary material for: Quercetin mitigates iron-induced cell death in chicken granulosa cell
Source: J Anim Sci Biotechnol. 2024 Dec 8;15:168. doi: 10.1186/s40104-024-01118-0 (PMC11625408; doi:10.1186/s40104-024-01118-0)
Supplement: Supplementary file 1 — Additional file 1: Fig. S1. Transmission electron micrographs of the atretic (A) and normal (B) follicles. The white triangles represented the lipid droplets; the black arrows showed mitochondria with structural defects; the white arrows showed the mitochondria with intact structures; the white dashed boxes delineate areas with a large accumulation of lipid droplets. Fig. S2 Iron levels of the atretic and normal follicles. Data are presented as mean ± SEM. *P < 0.05, **P< 0.01. Fig. S3 Morphology of granulosa cells under the electronic microscope. Fig. S4 GO and KEGG enrichment analysis of the differentially expressed genes between the erastin and DMSO groups. A–C Top pathways in GO enrichment analysis of the differentially expressed genes in the Erastin and DMSO groups. The y-axis represents GO terms, and the x-axis represents the significance level of the GO enrichment terms. D–F Top pathways in KEGG enrichment analysis of the differentially expressed genes in the Erastin group and DMSO groups. The x-axis showed the enrichment score, the y-axis showed the KEGG terms, bubble color indicates significance, and bubble size reflects the number of genes enriched in the pathway. Fig. S5 GO and KEGG enrichment analysis of the differentially expressed genes between the quercetin and DMSO groups. A–C Top pathways in GO enrichment analysis of the differentially expressed genes in Quercetin and DMSO groups. The y-axis represents the GO terms, and the x-axis represents the significance level of the GO enrichment terms. D–F Top pathways in KEGG enrichment analysis of the differentially expressed genes in Quercetin and DMSO groups. The x-axis represents the enrichment score, the y-axis represents KEGG terms, bubble color indicates significance, and bubble size reflects the number of genes enriched in the pathway. Fig. S6 RT-qPCR validation results of target genes filtered from sequencing data. Validation results of the target genes (ACSL4, SLC7A11, and TFRC) filtered from transc [file 40104_2024_1118_MOESM1_ESM.doc]

**Table S1**. The composition and nutritional values of the basal diet (% dry matter)

| **Ingredient** | **Ingredient Content (%)** | **Nutrient** | **Nutrient Content** |
| --- | --- | --- | --- |
| Corn (maize) | 64.00 | Digestible Energy (MJ/kg) | 11.32 |
| Soybean meal | 23.00 | Crude Protein (%) | 17.01 |
| Wheat bran | 5.00 | Lysine (%) | 0.85 |
| Rice bran | 2.00 | Methionine + Cysteine (%) | 0.61 |
| Calcium bicarbonate | 1.00 | Calcium (%) | 1.80 |
| Stone powder | 2.00 | Phosphorus (%) | 0.25 |
| * Premix | 3.00 |  |  |
| Total | 100.00 |  |  |

* The premix provided per kilogram of diet: VA 9600 IU, VB1 3.2 mg, VB2 8.5 mg, VB6 7.9 mg, VB12 0.15 mg, VD3 7500 IU, VK3 7.5 mg, pantothenic acid 35 mg, niacin 80 mg, biotin 7.0 mg, choline 1000 mg, folic acid 1.5 mg, Mn 125 mg, Zn 85 mg, Fe 108 mg, Cu 42 mg, I 0.42 mg, Se 0.26 mg, Co 0.08 mg, total protein (TP) 4560 mg, NaCl 3780 mg.

**Table S2**. Primers used for the quantitative real-time PCR (qRT-PCR)

| **Gene name** | **Accession Number** | **Sequence of primers (5’-3’)** |
| --- | --- | --- |
| *ACSL4* | XM_040700144.2 | F: TGAATGTGGAGCGTCGTA |
|  |  | R: AGGTCTGCTTGGCTGAAT |
| *GPX4* | NM_001346448.2 | F: AGAATGGCGGACGAGTGG |
|  |  | R: CAGATCGACGAGCTGAGTGTAA |
| *FTH1* | NM_205086.2 | F: GCCACCTCCATCTACGAC |
|  |  | R: TGCTCCTTCAGCCACTTC |
| *GAPDH* | NM_204305.2 | F: CGGATTTGGCCGTATTGG |
|  |  | R: GGTCACGCTCCTGGAAGAT |
| *Caspase 3* | NM_204725.2 | F: AAGGCTCCTGGTTTATTC |
|  |  | R: CTGCCACTCTGCGATTTA |
| *Bcl-2* | NM_205339.3 | F: CAGAGGGACTTCGCCCAGAT |
|  |  | R: GTTGCCGTACAATTCCACAAA |
| *PTGS2* | NM_001167718.2 | F: TGACCCACAAGGCACAAA |
|  |  | R: ACCAAGCCAAACACCTCC |
| *Caspase 9* | XM_046931415.1 | F: CGAAGGAGCAAGCACGACA |
|  |  | R: CAGGTTGGACTGGGATGGAC |
| *NF-κB* | XM_046915553.1 | F: GCAGGCAGAGGTGGTAGA |
|  |  | R: CAGCAGCAATCAGTAGTTTC |
| *IL-6* | NM_204628.2 | F: GAAATCCCTCCTCGCCAATC |
|  |  | R: CCCTCACGGTCTTCTCCATAAA |
| *TNF-α* | XM_046927265.1 | F: TACCCTGTCCCACAACCT |
|  |  | R: CAGAGCATCAACGCAAAA |
| *Nrf2* | XM_046921130.1 | F: AACGCTGAACCACCAATC |
|  |  | R: TTCCCAAACTTGCTCTAT |
| *SLC7A11* | XM_426289.7 | F: TTTGGGTCTATGAATGGTG |
|  |  | R: GAAGAGGCAGGTGAAGGA |

F = Forward primer；R = Reverse primer

**Table S3.** Antibodies used in this study

| **Antibody** | **Company** | **Dilution ratio** | **Assay** |
| --- | --- | --- | --- |
| anit-β-Tublin | Zenbio (Chengdu, China) | 1:1000 | Weatern Blot |
| anti-Bcl-2 | ABclonal (Wuhan, China) | 1:1000 | Weatern Blot |
| anti-caspase-3 | Zenbio (Chengdu, China) | 1:1000 | Weatern Blot |
| anti-caspase-9 | Bioss (Beijing, China) | 1:1000 | Weatern Blot |
| anti-ACSL4 | Affinity (Cincinnati, USA) | 1:1000 | Weatern Blot |
| anti-Nrf2 | Affinity (Cincinnati, USA) | 1:1000 | Weatern Blot |
| anti-GPX4 | Affinity (Cincinnati, USA) | 1:1000 | Weatern Blot |
| anti-FTH1 | Affinity (Cincinnati, USA) | 1:1000 | Weatern Blot |
| anti-NF-κB | Zenbio (Chengdu, China) | 1:1000 | Weatern Blot |
| anti-TNF-α | Zenbio (Chengdu, China) | 1:1000 | Weatern Blot |
| anti-IL-6 | Zenbio (Chengdu, China) | 1:1000 | Weatern Blot |
| anti-IL-10 | Zenbio (Chengdu, China) | 1:1000 | Weatern Blot |
| anti-FSHR | Zenbio (Chengdu, China) | 1:100 | Immunofluorescence |

**Table S4**. Comparison of the adipose sequencing data with the reference genome

| **Sample name** | **Total_reads** | **Total_map** | **Unique_map** | **Multi_map** |
| --- | --- | --- | --- | --- |
| D1 | 48074934 | 45782602(95.23%) | 42371556(88.14%) | 3411046(7.10%) |
| D2 | 48054960 | 45526642(94.74%) | 42144392(87.70%) | 3382250(7.04%) |
| D3 | 48251022 | 45856095(95.04%) | 42525507(88.13%) | 3330588(6.90%) |
| E1 | 48362220 | 45961402(95.04%) | 42872184(88.65%) | 3089218(6.39%) |
| E2 | 48150992 | 45916068(95.36%) | 42776422(88.84%) | 3139646(6.52%) |
| E3 | 48114042 | 45192515(93.93%) | 42190503(87.69%) | 3002012(6.24%) |
| Q1 | 48082424 | 45680786(95.01%) | 43205978(89.86%) | 2474808(5.15%) |
| Q2 | 48447164 | 46190660(95.34%) | 43629599(90.06%) | 2561061(5.29%) |
| Q3 | 48611956 | 45915753(94.45%) | 43305368(89.08%) | 2610385(5.37%) |

Note: D (DMSO group), E (Erastin group), Q (Quercetin group).

**Table S5**. Potential target genes that influenced ferroptosis among the differentially expressed genes between Quercetin and Erastin

| Gene | FoldChange | log2FoldChange | *P*-value |
| --- | --- | --- | --- |
| *TFRC* | 6.751 | 2.755 | 0.001 |
| *ACSL4* | 2.808 | 1.490 | 0.001 |
| *SLC7A11* | 0.322 | -1.634 | 0.001 |

Note: Take three decimal places.


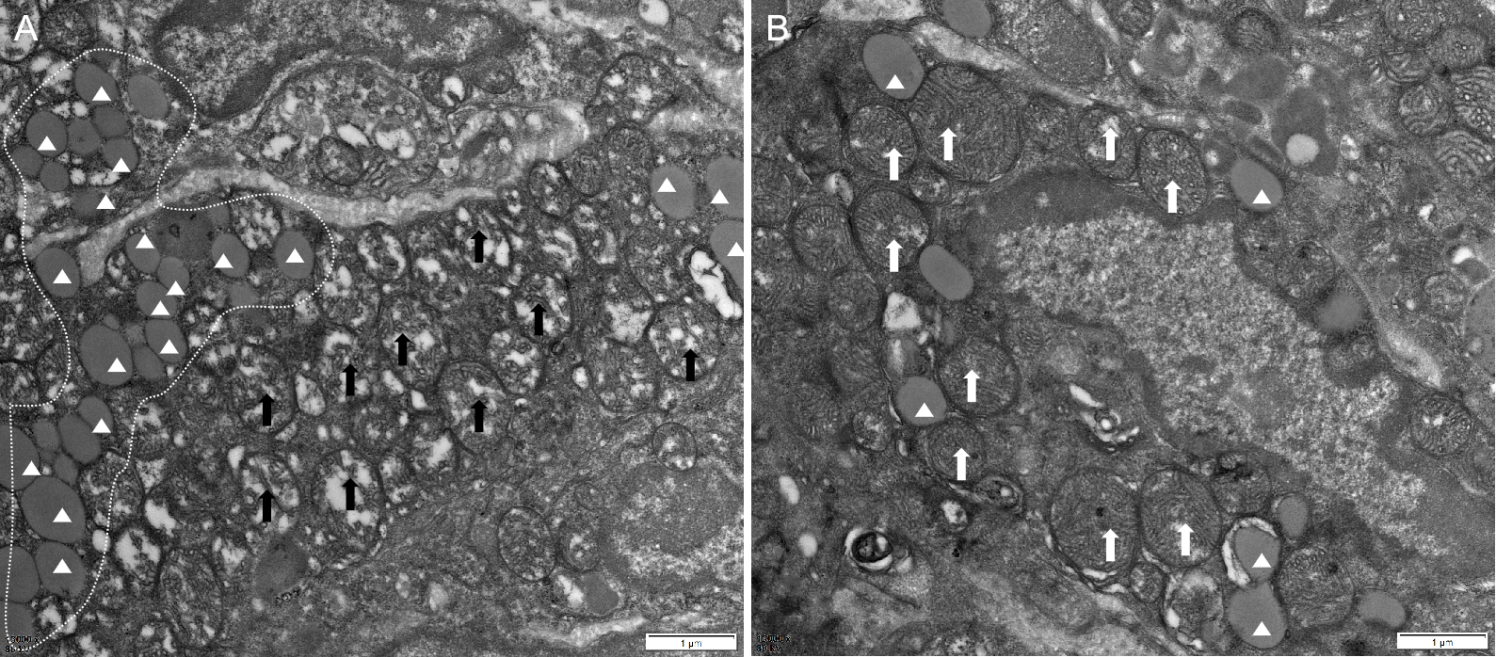


**Figure S1**. **Transmission electron micrographs of the atretic (A) and normal (B) follicles.**

The white triangles represented the lipid droplets; the black arrows showed mitochondria with structural defects; the white arrows showed the mitochondria with intact structures; the white dashed boxes delineate areas with a large accumulation of lipid droplets.


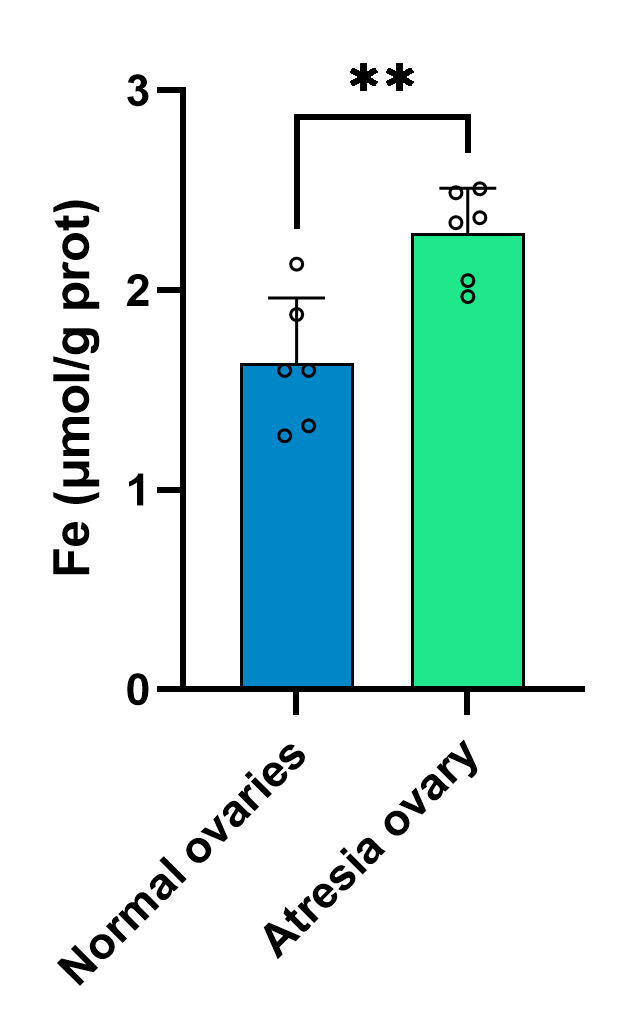


**Figure S2**. **Iron levels of the atretic and normal follicles.**

Data are presented as mean ± SEM. **P* < 0.05, ***P* < 0.01.

**Figure S3**. **Morphology of granulosa cells under the electronic microscope.**


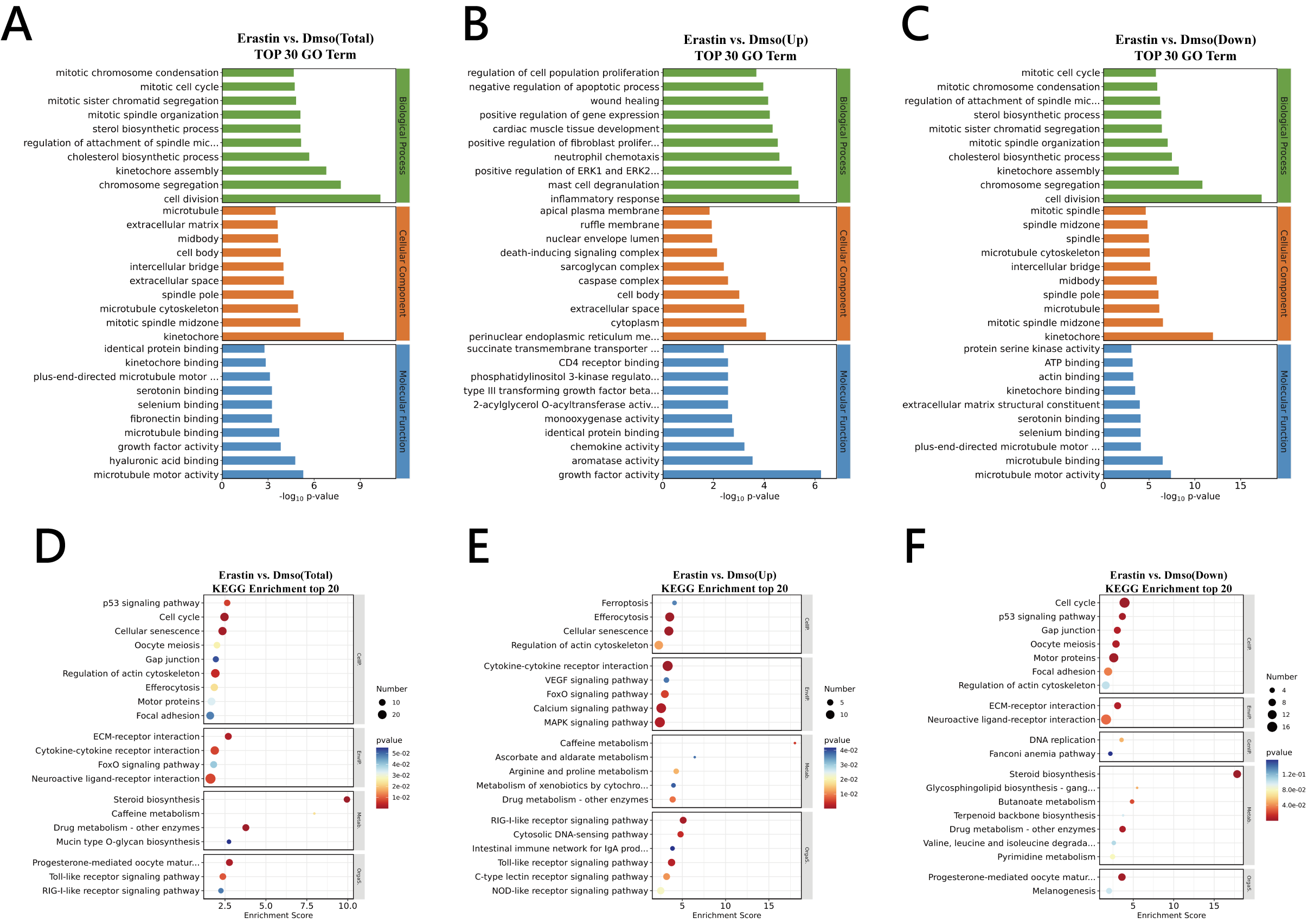


**Figure S4**. **GO (A-C) and KEGG (D-F) enrichment analysis of the differentially expressed genes between the Erastin and DMSO groups.**

(A-C) Top pathways in GO enrichment analysis of the differentially expressed genes in the Erastin and DMSO groups. The y-axis represents GO terms, and the x-axis represents the significance level of the GO enrichment terms. (D-F) Top pathways in KEGG enrichment analysis of the differentially expressed genes in the Erastin group and DMSO groups. The x-axis showed the enrichment score, the y-axis showed the KEGG terms, bubble color indicates significance, and bubble size reflects the number of genes enriched in the pathway.


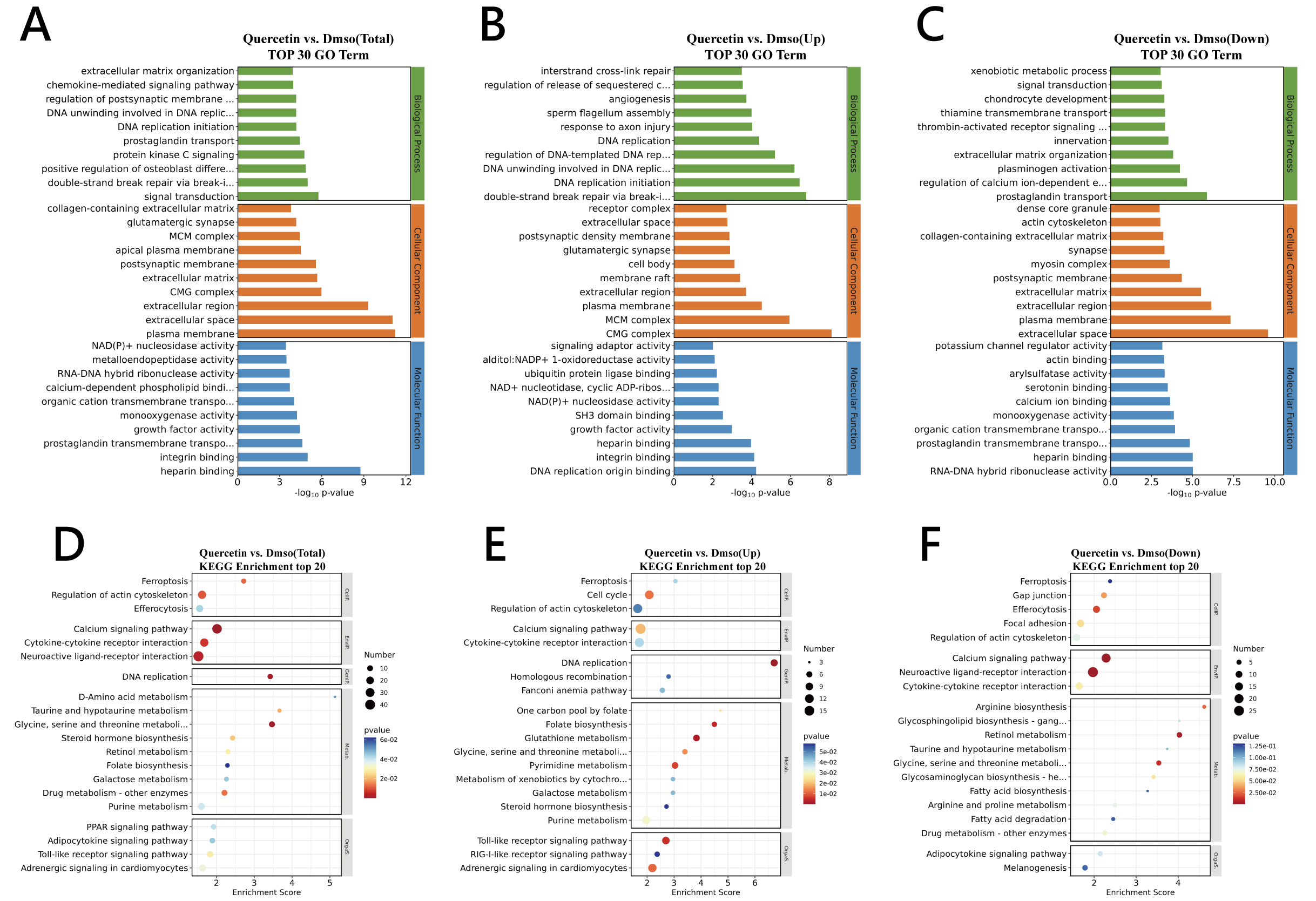


**Figure S5**. **GO (A-C) and KEGG (D-F) enrichment analysis of the differentially expressed genes between the Quercetin and DMSO groups.**

(A-C) Top pathways in GO enrichment analysis of the differentially expressed genes in Quercetin and DMSO groups. The y-axis represents the GO terms, and the x-axis represents the significance level of the GO enrichment terms. (D-F) Top pathways in KEGG enrichment analysis of the differentially expressed genes in Quercetin and DMSO groups. The x-axis represents the enrichment score, the y-axis represents KEGG terms, bubble color indicates significance, and bubble size reflects the number of genes enriched in the pathway.


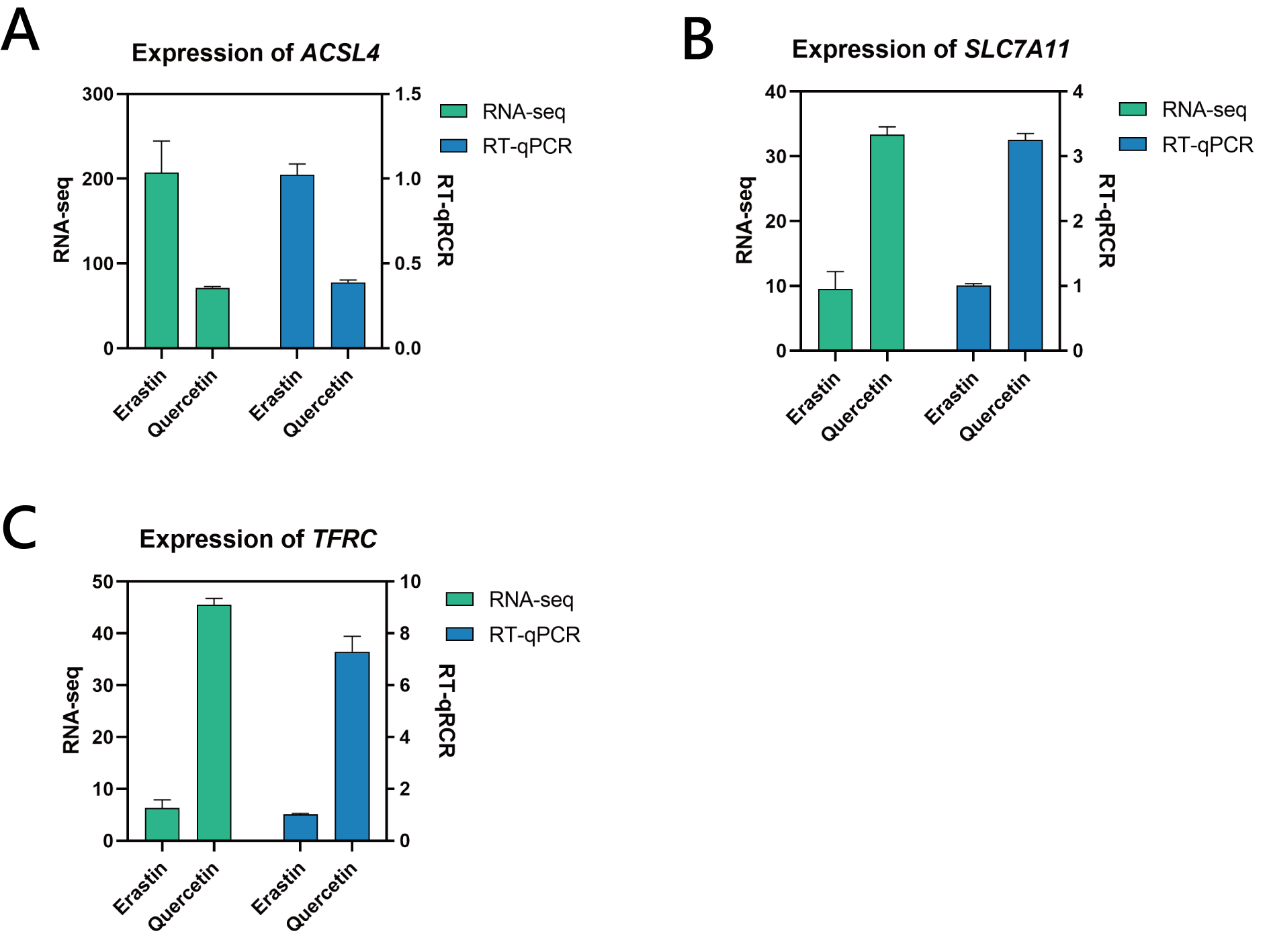


**Figure S6**. **RT-qPCR validation results of target genes filtered from sequencing data.**

Validation results of the target genes (*ACSL4*, *SLC7A11*, *TFRC*) filtered from transcriptome sequencing data were obtained via RT-qPCR. The RT-qPCR results were consistent with the transcriptome sequencing results.
